# Bacterial iron detoxification at the molecular level

**DOI:** 10.1074/jbc.REV120.007746

**Published:** 2020-10-12

**Authors:** Justin M. Bradley, Dimitry A. Svistunenko, Michael T. Wilson, Andrew M. Hemmings, Geoffrey R. Moore, Nick E. Le Brun

**Affiliations:** 1 Centre for Molecular and Structural Biochemistry, School of Chemistry, University of East Anglia, Norwich, United Kingdom; 2 School of Life Sciences, University of Essex, Colchester, United Kingdom; 3 Centre for Molecular and Structural Biochemistry, School of Biological Sciences, University of East Anglia, Norwich, United Kingdom

**Keywords:** iron regulation, Fur, DtxR, RirA, Irr, iron toxicity, iron storage, ferritin, bacterioferritin, Ftn, Dps, encapsulin, iron, iron metabolism, gene regulation, reactive oxygen species (ROS), Bfr, encapsulins

## Abstract

Iron is an essential micronutrient, and, in the case of bacteria, its availability is commonly a growth-limiting factor. However, correct functioning of cells requires that the labile pool of chelatable “free” iron be tightly regulated. Correct metalation of proteins requiring iron as a cofactor demands that such a readily accessible source of iron exist, but overaccumulation results in an oxidative burden that, if unchecked, would lead to cell death. The toxicity of iron stems from its potential to catalyze formation of reactive oxygen species that, in addition to causing damage to biological molecules, can also lead to the formation of reactive nitrogen species. To avoid iron-mediated oxidative stress, bacteria utilize iron-dependent global regulators to sense the iron status of the cell and regulate the expression of proteins involved in the acquisition, storage, and efflux of iron accordingly. Here, we survey the current understanding of the structure and mechanism of the important members of each of these classes of protein. Diversity in the details of iron homeostasis mechanisms reflect the differing nutritional stresses resulting from the wide variety of ecological niches that bacteria inhabit. However, in this review, we seek to highlight the similarities of iron homeostasis between different bacteria, while acknowledging important variations. In this way, we hope to illustrate how bacteria have evolved common approaches to overcome the dual problems of the insolubility and potential toxicity of iron.

A great deal of the biological importance of iron stems from facile redox transformations between the Fe^2+^ and Fe^3+^ oxidation states that underpin its function as a cofactor in many enzymes. Iron-containing proteins are grouped into three main classes. Iron-sulfur clusters are thought to represent the oldest class of iron-containing cofactors. They typically consist of 2–4 iron ions (although occasionally more) but occasionally also contain a heterometal, such as nickel or molybdenum, linked by inorganic sulfide and covalently attached to the protein via the thiol groups of cysteine residues. These versatile cofactors are involved in many processes, including respiration, photosynthesis, nitrogen fixation, hydrogen evolution, and the associated electron transfer chains (1). The simplest iron-containing cofactors are formed by the binding of discrete metal ion to sites composed from the side chains of histidine and/or the carboxylates aspartate and glutamate. These are principally employed to harness the oxidizing power of O_2_ for processes such as DNA synthesis and methane oxidation (2). Heme is formed by the incorporation of iron into the tetrapyrrole protoporphyrin IX. This chemically versatile cofactor is critical in many processes, including respiration, cycling of nitrogen, and sulfur and detoxification reactions in addition to also supporting electron transfer (3–5). As a result of this versatility, the demand for iron is large in most organisms, including the majority of bacteria, with up to 25% of the proteome binding iron in some form (6).

However, the same redox chemistry required for these roles (Reaction 1 and the Fenton reaction, Reaction 2) allows iron to catalyze the Haber–Weiss reaction (Reaction 3).

$${\text{Fe}}^{3+}+{\text{O}}_{2}^{\overset{¯}{˙}}↔{\text{Fe}}^{2+}+{\text{O}}_{2}$$

$${\text{Fe}}^{2+}+{\text{H}}_{2}{\text{O}}_{2}↔{\text{Fe}}^{3+}{+}^{-}\text{OH}{+}^{•}\text{OH}$$

$${\text{O}}_{2}^{\overset{¯}{˙}}+{\text{H}}_{2}{\text{O}}_{2}{↔}^{-}\text{OH}{+}^{•}\text{OH}+{\text{O}}_{2}$$

Reactions 1–3

The resulting hydroxyl radicals (^•^OH) are highly reactive, causing damage to lipids, proteins, carbohydrates, and nucleic acids (7). Superoxide (${\text{O}}_{2}^{\overset{¯}{˙}}$) and hydrogen peroxide (H_2_O_2_) are produced as by-products of aerobic respiration (8), and, therefore, any aerobically respiring organism faces the requirement not only to detoxify ROS but also to strictly regulate the concentration of iron in any form able to catalyze the Haber–Weiss reaction. This need is particularly acute in the case of bacteria because, in addition to endogenously produced ROS, they are often subjected to assault by ROS produced either by competitors in the environment or in phagocytes produced by the immune system of hosts during infection (9).

Nitric oxide is known to play an important role as a signaling molecule in biological systems but is also produced in elevated concentrations for defense or competition in a similar manner to ROS. Combination of nitric oxide with superoxide generates the peroxynitrite ion that is susceptible to further oxidation to either nitrogen dioxide or dinitrogen trioxide. Collectively, these RNS can cause damage to nucleic acids and modify the side chains of amino acids such that protein structure and function are impaired (9). Furthermore, both ROS and RNS are known to lead to breakdown of iron-sulfur clusters, resulting in the displacement of iron from the cofactor. Thus, iron homeostasis and the generation of ROS and RNS are intimately connected, as are the regulatory networks for their management within bacterial cells.

## Sensing of iron and regulation of genes involved in iron uptake/homeostasis

**Figure 1.**
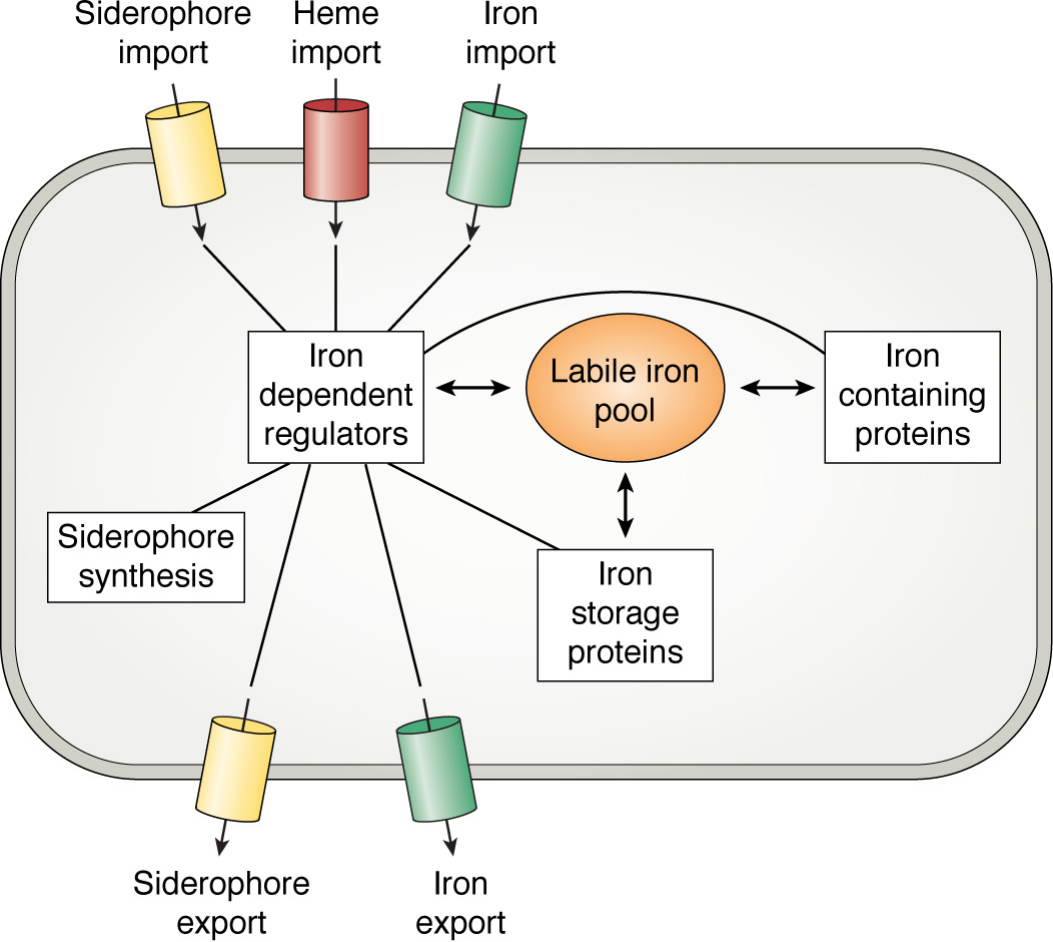
**Routes of iron trafficking in bacterial cells.** *Heavy arrows* depict intracellular movement of iron, *light arrows* show the movement of iron or iron-bearing compounds across the cell membrane, and *lines* connect the transcriptional regulators to systems under their control. When the concentration of the labile iron pool increases, iron, or an iron-containing group, binds to the transcriptional regulator. This leads to down-regulation of processes such as siderophore synthesis, export of apo-siderophores, import of Fe^3+^-siderophores, heme import, and Fe^2+^ uptake systems. Simultaneously, expression of iron-containing and iron storage proteins is up-regulated together, occasionally, with iron efflux pumps. Reduction in the labile iron pool leads to dissociation of iron/iron-containing groups from the regulators, resulting in the opposite transcriptional responses.

When considering the iron status of cells, it is important to distinguish between the quota, which is the total iron content of the cell, and that subset of the quota that is kinetically available for insertion into proteins and molecular cofactors, referred to as the “labile iron pool” (10). The majority of the latter is likely in the Fe^2+^ oxidation state and coordinated by small molecules, such as low-molecular weight thiols (11, 12). This represents the fraction of the quota available to fulfill metabolic requirement, but also that with the potential to catalyze unwanted ROS and RNS formation. Therefore, the first requirement of any regulatory system for iron homeostasis is the ability to sense the concentration of the labile iron pool across the physiologically relevant range, 1–10 μm according to most estimates (13–15). As one might expect, this is achieved by transcriptional regulators whose affinities for target DNA are modulated by either binding directly to iron or by the binding of iron-dependent prosthetic groups. Often these are global regulators, controlling the expression of a great many genes, including those involved in the biosynthesis and import of siderophores, import of ferrous iron, and the storage and/or efflux of iron present in excess of cellular requirements. This balancing of metal trafficking to fulfill nutritional requirements while suppressing potential toxicity, shown schematically in Fig. 1, is termed “nutritional passivation” and is a common strategy that extends beyond iron metabolism (16).

### Iron sensing by Fur

Members of the Fur (ferric uptake regulator) superfamily are the most widespread transcriptional regulators controlling iron homeostasis in bacteria. The first member of the Fur family was identified in *Escherichia coli* some 35 years ago (17) and, as the name suggests, was reported to regulate the intake of Fe^3+^ into the cell. This is achieved by the binding of the protein to “Fur boxes,” AT-rich binding sites upstream of the regulated genes with the consensus sequence 5′-GATAATGATAATCATTATC-3′. It has been argued that the Fur box should be considered a 21-bp fragment containing two overlapping 7-1-7 inverted repeats that each bind a Fur dimer.

**Scheme 1.**
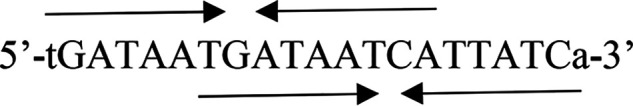


These are positioned such that the two copies of Fur bind to opposite faces of the DNA helix (18). Binding of Fur occludes access of RNA polymerase, thus repressing transcription of the responsive genes (19). However, despite the great deal of research effort directed at members of the Fur superfamily, an understanding of these processes at the molecular level has only recently been achieved.

Despite reports of both monomeric (20) and higher oligomeric (21) forms of Fur detected in solution, the physiologically relevant form of the protein is thought to be the homodimer. This is stabilized by a large buried interface between C-terminal dimerization domains (22) and, in most cases, the binding of a structural Zn^2+^ (23) ion by four conserved Cys residues (24). Occupancy of this structural site (S1) is required, but not sufficient, for DNA binding. The Fur family exhibits some structural variation, and in certain examples, the dimerization domain harbors a second structural site ligated by His and Glu residues (25). The dimerization domain is connected to the N-terminal DNA-binding domain via a flexible hinge region containing a regulatory site comprising His and Glu side chains that binds Fe^2+^ with a reported dissociation constant, *K_d_*, of ∼1 μm when determined *in vitro* (26). Whereas the regulatory site has been demonstrated to bind other di- and trivalent metals, it is thought that only Fe^2+^ is present at the concentration required to activate the protein *in vivo*. Occupancy of this site induces a rotation of the DNA-binding domain relative to the dimerization domain, creating an increased void area between the two DNA-binding domains such that they are able to accommodate dsDNA (25). It is thought that this conformational change forms the molecular basis of the increased affinity of Fur for DNA *in vitro* under elevated concentrations of the regulatory metal. *In vitro* studies utilizing gel-shift methods report *K_d_* values of ∼10 nm for complex formation between activated Fur and target DNA sequences (23).

**Figure 2.**
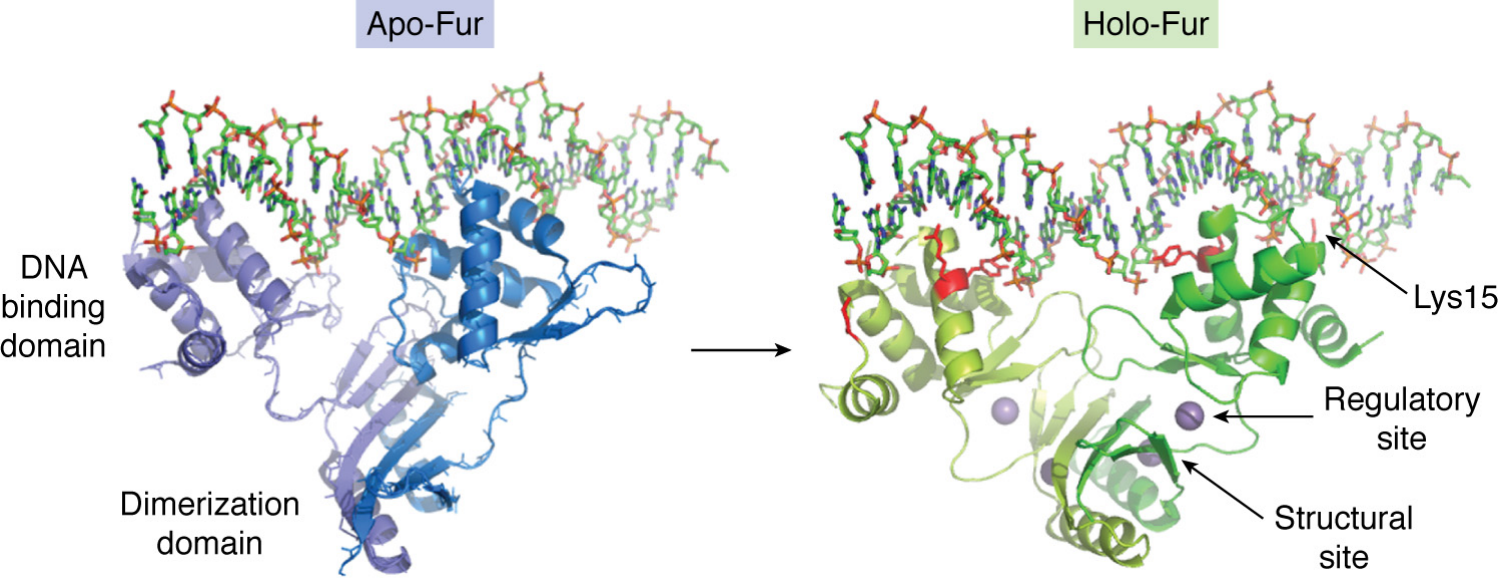
**Domain movements induced by the binding of divalent metals to Fur.** Binding of divalent metal ions to the regulatory site of Fur induces a rotation of the DNA-binding domain relative to the dimerization domain, bringing the DNA recognition helices into more favorable alignment for binding to the Fur box. Residues Lys-15, Tyr-56, and Arg-57, which form favorable interactions with the nucleotide, are *highlighted* in *red*. Reproduced from PDB depositions 4RAY and 4RB1 (27).

The recently reported crystal structure of *Magnetospirillum gryphiswaldense* Fur (27) in complex with DNA has provided insight into the molecular basis for recognition of Fur-binding sites (Fig. 2). The AT-rich composition of the Fur box results in a narrowing of the minor groove and consequent increase in negative charge density from the phosphate backbone that persists upon repressor binding. This facilitates shape recognition by Fur via a favorable electrostatic interaction between a conserved lysine residue (Lys-15 in *M. gryphiswaldense* Fur numbering) and the minor groove. More specific interactions with bases in the major groove are facilitated by the rotation of the DNA-binding domains induced by metal binding at the regulatory site. This involves van der Waals interactions between Tyr-56 and consecutive thymine bases in the target sequences and hydrogen bonding between the guanidinium group of Arg-57 and the O6 and N7 atoms of a conserved guanine. A recent report suggests that Fur DNA binding can be tuned by protein-protein interactions (28), in addition to the long-recognized effect of iron binding. EIIA^Ntr^, a component of the nitrogen metabolic phosphotransferase system, was shown to affect expression of Fur-regulated genes. *In vitro* gel shift measurements showed that this arises from formation of a protein-protein complex that lowers the affinity of holo-Fur for DNA. Consequently, repression of Fur-regulated genes requires a greater cytoplasmic Fe^2+^ concentration when EIIA^Ntr^ is present. The *K_d_* for the Fur-EIIA^Ntr^ complex has not yet been determined; nor has the increase in *K_d_* of the Fur-DNA complex in the presence of EIIA^Ntr^.

In addition to the classic gene repression mechanism described above, Fur has been shown to act as an activator of gene expression, both directly (29–32) and indirectly (33). Direct activation occurs through binding in the promoter region (29–32), whereas indirect regulation occurs via interaction with the noncoding RNA RhyB (see the Iron storage in bacteria section below) (34), by the displacement of histone-like proteins (35), or by blocking the binding of a second repressor (36). Regulation of gene expression by apo-Fur has also been demonstrated in a limited number of cases (37, 38), and genome-wide studies have demonstrated the Fur regulon to encompass dozens of transcription units, containing >100 genes in some cases (33, 37, 39–41). It is now apparent that Fur-like proteins constitute a superfamily with members identified that are responsive to other metals (Mur, the manganese uptake regulator (42), and Zur (43), the zinc uptake regulator) and to peroxide-induced oxidative stress (Per) (23).

Genes identified as being regulated by Fur, such as that in *E. coli*, include those encoding iron-uptake systems, such as *fhu*, *fec*, and *feo*; the *suf* iron-sulfur cluster assembly system; iron-sulfur–containing proteins, such as *fumA*, *acnA*, *acnB*, and *nuo*; the iron-containing superoxide dismutase *sodB*; and the iron storage proteins *bfr* and *ftnA* (see below). Consistent with its role as a repressor of iron import systems, the transcriptional response of a Fur deletion mutant is similar to that evoked by iron limitation, even under iron-replete conditions. This inability to correctly sense the iron status of the cell has been demonstrated to result in an increase in ROS production (44), suggesting that, in contrast to some other metals, cellular storage and efflux mechanisms are unable to compensate for the resulting elevated concentration of the labile iron pool. Fur has been shown to be involved in the remodeling of cell metabolism away from iron-containing enzymes, management of ROS, and reconfiguration of the cell membrane to protect against antibiotic attack, in addition to controlling cellular iron homeostasis (10, 33, 45).

### Iron sensing by DtxR/IdeR

Proteins of the DtxR/IdeR (diphtheria toxin repressor/iron-dependent regulator) family are the global transcriptional regulators controlling iron uptake in GC-rich Gram-positive bacteria (46). Indeed, DtxR was first identified as an iron-dependent repressor of virulence factor expression in *Corynebacterium diphtheriae*, and it is from this activity that the name derives (47). Much effort has been devoted to the study of this group of bacteria as they include important human pathogens such as *C. diphtheriae* itself, *Mycobacterium tuberculosis*, and *Staphylococcus aureus* and antibiotic producers such as *Streptomyces*. This included the demonstration that DtxR also regulates iron uptake in *C. diphtheriae* and the identification of homologues in other organisms.

Proteins of this family exhibit similarities to Fur; they act primarily as repressors of transcription by occluding binding of RNA polymerase (48, 49) but recognize a consensus sequence with greater GC content than that of Fur: 5′-TTAGGTTAGCCTAACCTAA-3′ (50). The homodimers harbor multiple metal-binding sites and undergo conformational change upon binding Fe^2+^ as corepressor. In the metal-bound active form, dsDNA binds between two helix-turn-helix (HTH) N-terminal DNA-binding domains that are linked via dimerization domains (51, 52). *In vitro* DNA affinity of Fe^2+^-sensing DtxR proteins is also activated by noncognate divalent metal ions such as Ni^2+^, Co^2+^, Mn^2+^, and Cd^2+^. Ni^2+^ and Fe^2+^ bind DtxR with the highest affinity, *K_d_* being around 1 μm (53, 54). However, distinct from Fur, these proteins also contain an SH3-like domain of unknown function as a C-terminal extension (52). They also differ in the molecular contacts leading to recognition of target DNA and the nature of the conformational change induced by binding of the regulatory metal.

**Figure 3.**
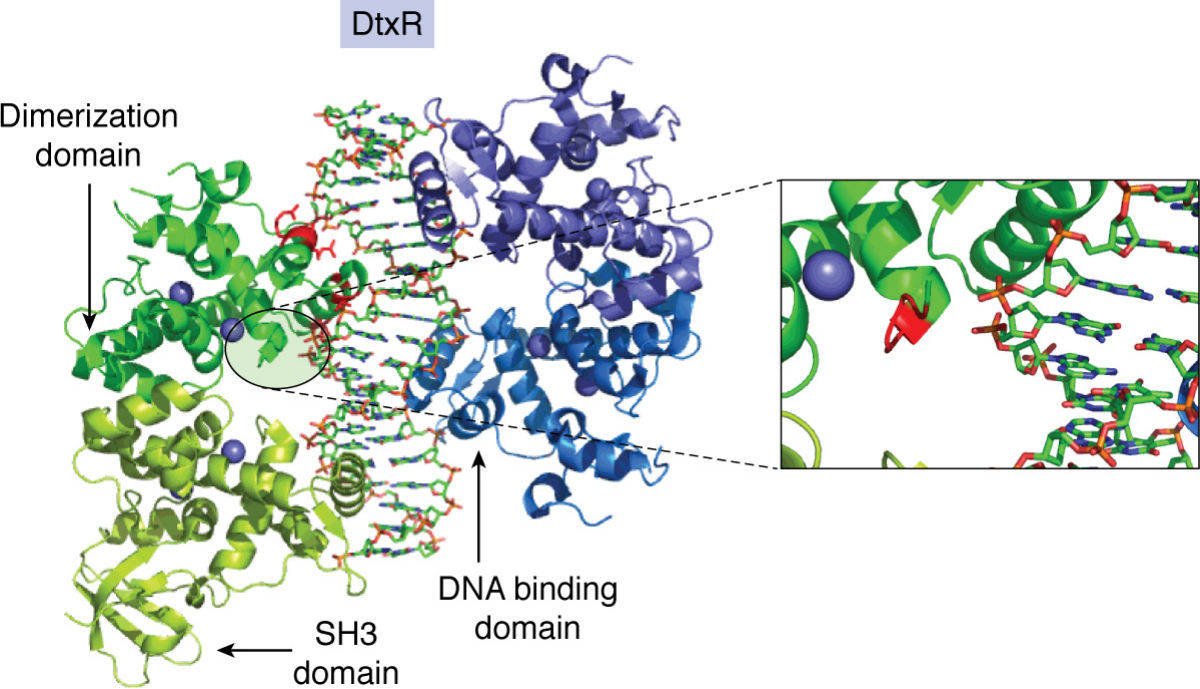
**Binding of DtxR to a 21-base pair model oligonucleotide.** Identical DtxR dimers bind to opposite faces of the nucleotide, but only one of the four SH3-like domains is resolved crystallographically. The *inset* shows the N-terminal region of the protein with residues 3–6 *highlighted* in *red*. Upon binding of the regulatory metal ion, the highlighted region undergoes a helix-to-coil transition that relieves what would otherwise be an unfavorable steric interaction between protein and DNA. Also *highlighted* in *red* are residues Arg-27, Ala-28, Arg-29, Thr-40, Ser-42, Arg-47, Arg-50, and Arg-60, which form favorable interactions with the nucleotide. Reproduced using PDB deposition 1C0W (52).

Structures of DtxR in complex with DNA were available before those of Fur and revealed two homodimers bound to each nucleotide fragment (51) (Fig. 3). Each of the monomers harbors two metal-binding sites (presumed to be iron *in vivo*), and, in further analogy to Fur, binding of divalent metal to the high-affinity ancillary site imparts stability to the protein fold, whereas affinity for target DNA sequences is increased by the occupancy of the lower-affinity primary site (46, 55, 56). However, in contrast to Fur, occupancy of the primary metal-binding site results in only a small rotation of the DNA-binding domains relative to the dimerization domains (52). Comparison of apo- and holo-structures of DtxR suggests that metal ion binding induces a helix-to-coil transition in the six N-terminal residues (51, 56) and relieves what would, in the apoprotein, be an unfavorable steric interaction with DNA. This, together with a small “caliper-like” movement of the N-terminal domains, which brings them into better alignment with the major groove, results in the increased DNA affinity for the holo form of the repressor over the apo form (51). Residues 27–50 make up the helix-turn-helix DNA-binding motif containing helices B and C. Each monomer contributes a total of nine favorable interactions with nucleotide phosphate groups: Arg-27, Ala-28, and Arg-29 of helix B and Thr-40, Ser-42, Arg-47, and Arg-50 of helix C together with Glu-36 and Ser-37 of the intervening loop. In further contrast to Fur, formation of the protein-DNA complex causes distortion of the nucleotide from the *B*-form conformation. This results in the opening up of the minor groove such that it can accommodate the guanidinium group of Arg-60, located on the loop 57–61 connecting two strands of an antiparallel β-sheet (51).

Examples of the DtxR/IdeR family lacking the C-terminal SH3-like domain have been reported, but these are not responsive to Fe^2+^ *in vivo* (57, 58). Given the recent discovery that DNA binding by Fur is modulated by formation of a complex with EIIA^Ntr^, it is possible that the SH3 domain modulates the iron response of DtxR via protein-protein interactions. The suite of genes regulated by DtxR includes those involved in siderophore production and translocation, heme degradation, Fe^2+^ import, iron-sulfur cluster assembly, and iron storage (59), demonstrating similar regulatory activity to Fur despite there being no evolutionary link between the two protein families.

### Iron sensing by RirA and Irr

The genomes of the α-proteobacteria contain homologues of Fur, but where these have been characterized, they have been shown either to have a diminished role in iron regulation compared with other examples of Fur or to be responsive to other metals, such as Mn^2+^ (60, 61). Global regulation of iron is performed by two novel transcriptional regulators found, with few exceptions, only within the α proteobacteria (62): iron response regulator (Irr) (63) and rhizobial iron regulator A (RirA) (64). These are currently less well-characterized than either Fur or DtxR, with no crystal structures of either the proteins or protein/DNA complexes available to date. However, significant progress in understanding these proteins has been made recently, and both are known to sense the availability of intracellular iron not by binding the metal itself, but instead by binding iron-containing prosthetic groups.

RirA is unique among bacterial iron-sensing transcriptional regulators in that it belongs to the Rrf2 family (64). As with many members of this family, affinity of RirA for DNA is modulated by the binding of an iron-sulfur cluster (65). Again, the protein exists as a homodimer in solution, and homology modeling based on the recently reported structures of other Rrf2 regulators predicts that each monomer contains a DNA-binding domain, featuring a winged helix-turn-helix motif, connected to a dimerization helix via a loop containing three conserved Cys residues (66).

Under iron-replete conditions, the protein contains a [4Fe-4S]^2+^ cluster, coordinated by the three conserved Cys residues (67) (with a likely additional, but unknown, ligand), and binds to *cis*-acting iron-responsive operator sequences (68) (IRO boxes) in the promoter region of genes involved in iron uptake acting as a repressor of transcription in a manner analogous to Fur and DtxR. The apoprotein lacks any specific high-affinity interaction with DNA *in vitro*, whereas a meta-stable [2Fe-2S]^2+^ cluster–containing form has been shown to exhibit intermediate binding affinity (69). RirA has also been shown to promote transcription of genes (70, 71), including those involved in iron storage under iron-replete conditions, via an indirect mechanism involving small noncoding RNA (72) in analogy to Fur.

Recent *in vitro* characterization of RirA from *Rhizobium leguminosarum* demonstrated that iron sensing occurs via a reversible dissociation of a labile Fe^2+^ ion from the [4Fe-4S]^2+^ cluster, with a *K_d_* of 3 μm (66). Under iron-replete conditions, the cluster remains stable in the [4Fe-4S]^2+^ form. However, when iron is limiting, competition for the labile iron increases, yielding a [3Fe-4S]^0^ cluster intermediate that is unstable to further breakdown to the apo-form, via a [2Fe-2S] form (as well as several other intermediates). Under low iron and in the presence of O_2_, accelerated degradation to apo-RirA occurs. This results initially from the oxidation of the [3Fe-4S]^0^ intermediate to a less stable [3Fe-4S]^1+^ form and is subsequently mediated by the oxidation of cluster sulfides. This susceptibility to O_2_-mediated iron and sulfur oxidation is thought to underpin a dual Fe^2+^- and O_2_-sensing role. RirA has been demonstrated to regulate iron-sulfur cluster biogenesis in *R. leguminosarum*, and O_2_ sensing by RirA may be important to ensure adequate cellular supply of iron-sulfur clusters under aerobic conditions even when iron is replete. An as yet unknown regulatory mechanism prevents up-regulation of iron-uptake systems under these conditions (66).

Whereas RirA is restricted to the order *rhizobiales*, Irr is widely distributed among the α-proteobacteria (72, 73). The protein is a homologue of Fur but senses the iron status of the cell not by binding Fe^2+^ from the free iron pool, but the iron-containing prosthetic group heme (43). Due to the insolubility and potential cytotoxicity of heme, cells are unlikely to contain a “free heme pool” akin to that of Fe^2+^. Rather, it is thought that Irr is associated with ferrochelatase (74), the enzyme responsible for insertion of iron into protoporphyrin IX in the final step of heme biogenesis, and acquires the prosthetic group directly from it. Apo-Irr binds to iron control element (67, 75) sequences (ICE boxes) that are upstream of regulated genes and, like other Fur proteins, can act directly either as a repressor or an activator, depending on the location of the ICE sequence (75). However, in the case of Irr, direct activation of regulated genes is far more common than for either Fur or DtxR. In further contrast to other examples of the Fur superfamily, Irr only binds to ICE sequences in the absence of its co-regulator. All examples characterized to date contain two heme-binding sites. One of these is a conserved H*X*H motif (76, 77), but studies have revealed significant diversity in the nature of the other. Possibly related to this, the mechanism by which derepression occurs appears to differ markedly between members of the *rhizobiales* in which RirA also acts as an iron-responsive global regulator and other α-proteobacteria in which Irr is the only protein fulfilling this function. The best-characterized examples are the Irr proteins from *R. leguminosarum* (belonging to the former class) and *Bradyrhizobium japonicum* (from the latter).

In organisms such as *R. leguminosarum*, *Agrobacterium tumefaciens* (78), and *Ensifer meliloti* (72), Irr forms part of a regulatory network involving RirA among other factors. These networks are interlinked, with Irr controlling expression of RirA while the two proteins regulate iron homeostasis in an antiparallel manner. Under high-iron conditions, RirA represses the expression of iron uptake systems, whereas in low iron Irr represses the expression of iron storage systems but also RirA, thereby assisting in derepression of RirA-regulated genes. The proteins are dimeric in solution, and loss of DNA-binding affinity is associated with the binding of heme at the H*X*H motif located close to the interface between the monomers (77). Disruption of this heme-binding motif by mutagenesis led not only to the abolition of heme binding but also high-affinity binding of DNA by the apoprotein, thereby demonstrating the importance of this motif for the recognition of ICE box sequences. These observations led to a model in which a conformational change in the H*X*H motif upon binding of heme forms the molecular basis of the loss of DNA affinity. However, the detail of any such conformational change at the atomic level remains to be elucidated. Whereas the regulatory role, if any, of the second heme-binding site remains unclear, its occupancy has been shown to modulate the oligomeric state of the protein *in vitro* (79).

In organisms such as *B. japonicum*, in which Irr is the only global regulator of iron homeostasis, regulation is achieved via a different mechanism. These proteins have an H*X*H heme-binding motif similar to that identified in Irr from *Rhizobiales* (76, 80), but this site preferentially binds heme with iron in the Fe^2+^ oxidation state. Furthermore, the binding of heme does not affect the affinity of the protein for DNA binding; rather, the protein has been shown to be conditionally stable with degradation initiated by the binding of ferric heme at a second site, the heme regulatory motif. On binding to this site, the heme iron is five-coordinate with the sulfur of a cysteine residue providing the axial ligand. Pulsed radiolysis studies demonstrated a ligand switch to axial ligation by histidine upon reduction followed by binding of O_2_ under aerobic conditions (81). This has led to the suggestion of a ROS-mediated pathway for *B. japonicum* Irr degradation in the presence of heme. The available data indicate that heme binding to both sites of *B. japonicum* Irr is required for efficient degradation of the protein. The heme regulatory motif is not limited to *B. japonicum* Irr, having also been identified in Irr proteins from *Nitrobacter*, *Xanthobacter*, and *Magnetospirrilum* (61), suggesting a similar mechanism of iron regulation in these organisms.

In the absence of both of its substrates, ferrochelatase binds Irr with high affinity, thereby competing with DNA binding and alleviating regulatory activity. However, binding of protoporphyrin IX to ferrochelatase causes dissociation of its complex with Irr. Therefore, when the rate of heme synthesis outstrips the availability of iron, Irr is released, down-regulating iron-dependent biosynthetic pathways and activating genes involved in iron acquisition. Once the concentration of Fe^2+^ in the labile iron pool increases sufficiently such that metalation of protoporphyrin IX is coordinated with its synthesis, heme is inserted into Irr, targeting the protein for oxidative degradation and therefore ensuring that regulatory activity is abrogated. It is thought that this system of regulation allows the rate of iron uptake to be matched to metabolic need under varying conditions rather than simply maintaining the labile iron pool at a concentration determined by the affinity of Fe^2+^ for the transcriptional regulator (74).

## Iron acquisition by bacteria

Despite its natural abundance in the earth's crust, iron is often a growth-limiting micronutrient for bacteria due to the insolubility of the Fe^3+^ ion at neutral pH, which limits the dissolved iron concentration to 1.4 × 10^−9^ m under aerobic conditions (82). To counter the low bioavailability of iron in many environments, bacteria have evolved high-affinity iron acquisition pathways. Whereas these are often targeted by host immune systems or competing bacteria to limit growth (83), they are also under the control of the global regulators described above to enable expression to be repressed should iron availability exceed cellular requirement (84). Iron uptake in bacteria has been extensively studied with the ultimate aim of preventing infection by targeting iron metabolism. Here we survey the main features while referring the interested reader to several recent reviews (85–88).

### Siderophore-mediated iron uptake

The most widely distributed iron acquisition strategy under aerobic conditions is the secretion of siderophores (89). These are small-molecule chelators (150–2000 Da) (90) with high affinity for Fe^3+^ (*K_d_* in the range 10^−20^ to 10^−49^ m) that acquire iron from the extracellular environment (85). Over 500 examples have been characterized to date falling in to three main classes, the catechols, hydroxamates, and α-hydroxycarboxylates, defined according to the nature of the iron-ligating moiety (89). Examples containing more than one of the aforementioned iron-ligating groups are termed mixed siderophores.

Siderophore synthesis is nonribosomal but occurs in the cytoplasm, meaning that in Gram-negative bacteria, their export and, in most cases, utilization of the sequestered iron requires transport across both the cytoplasmic and periplasmic membranes. There appears to be the greatest diversity in the proteins involved in the export across the cytoplasmic membrane, with examples belonging to both the ABC transporter (91) and major facilitator superfamily (MFS) (92) classes reported. Export across the outer membrane is mediated by TolC-like efflux pumps (93).

**Figure 4.**
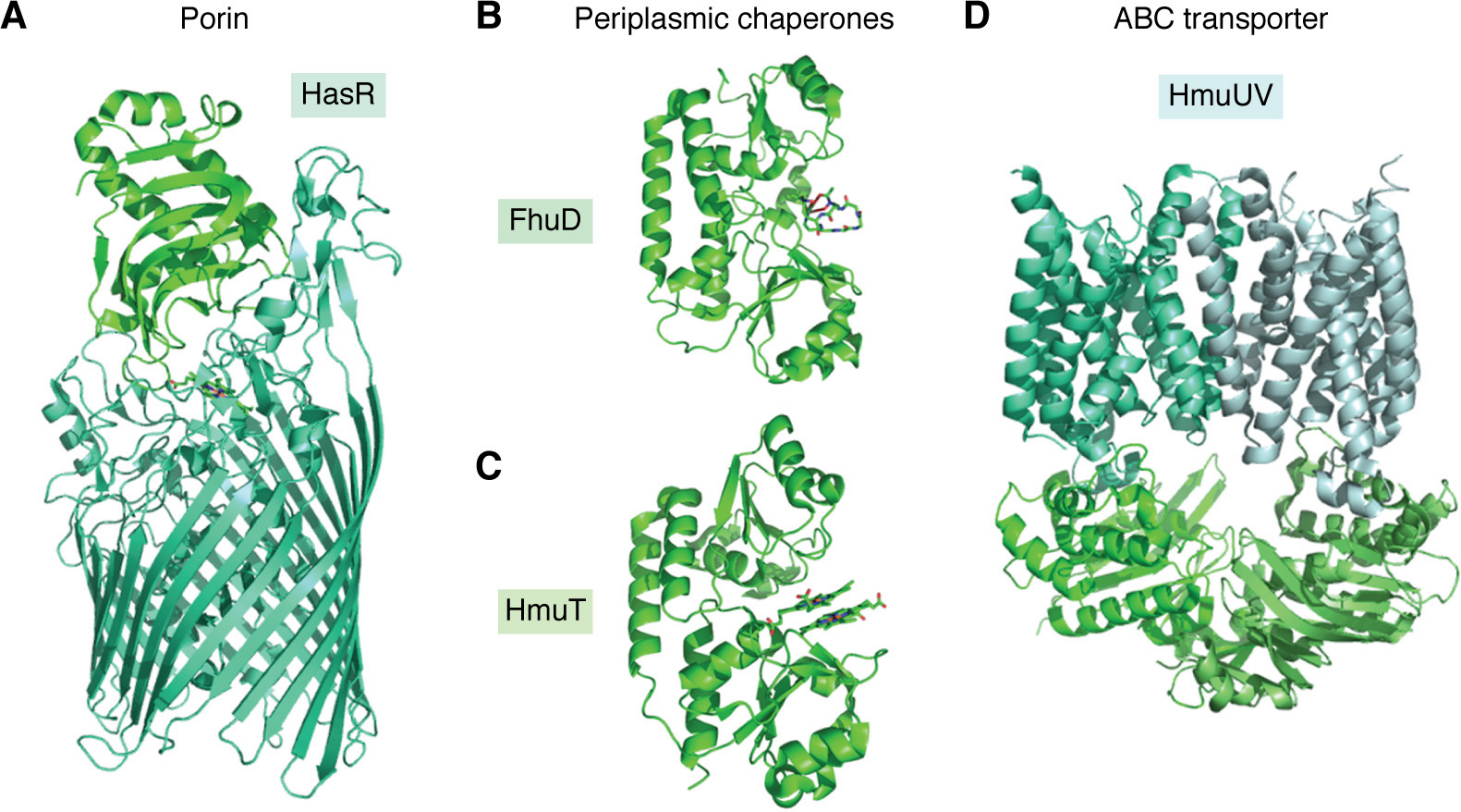
**Structures of representative proteins involved in bacterial iron acquisition.** *A*, HasR, a β-barrel porin involved in transport of heme across the periplasmic membrane in complex with HasA. The importers of siderophores exhibit very similar topology. Also shown are chaperone proteins FhuD (*B*) and HmuT (*C*), which shuttle siderophores and heme, respectively, across the periplasmic space as well as the ABC transporter HmuUV (*D*), which transports heme across the cytoplasmic membrane. ABC transporters involved in siderophore transport exhibit similar topology. Reproduced from PDB depositions 3CSL (101), 1EFD (223), 3NU1 (224), and 4G1U (225).

Once secreted from the cell, siderophores acquire iron either by outcompeting host proteins, such as transferrin, or by the solubilization of Fe^3+^ from iron-containing minerals. Import across the outer membrane is mediated by porins (Fig. 4*A*) composed of 22-stranded β-barrels and an extracellular facing “plug” domain that binds the iron-loaded siderophore with high (typically nanomolar) affinity. The TonB/ExbBD energy-transducing complex spans the periplasmic space and connects the porin to the cytoplasmic membrane potential, allowing active transport of the substrate.

Once internalized, periplasmic binding proteins (Fig. 4*B*) act as chaperones delivering the ferric siderophore complex to the cytoplasmic membrane. Here ABC transporters (Fig. 4*D*) couple transport across the inner membrane to ATP hydrolysis. Many bacteria are able to utilize multiple siderophores to satisfy their requirement for iron, including “xenosiderophores,” those that the organism is unable to synthesize but can internalize and extract iron from (94). A general trend is that the outer-membrane porins show specificity for their cognate siderophore, whereas the inner-membrane ABC transporters have greater flexibility in substrates tolerated. Therefore, the genomes of Gram-negative bacteria encode a greater number of outer-membrane porins for siderophore uptake than ABC transporters dedicated to the same task (95).

Once the loaded siderophore has been translocated to the cytoplasm, the iron is typically released via reduction to Fe^2+^ (95), for which the chelators have lower affinity. A possible exception to this are the hexadentate triscatechelates, which form the most stable Fe^3+^ complexes of all siderophores, stabilizing this oxidation state to such an extent that the midpoint of the Fe^3+^/Fe^2+^ couple is in the range −600 to −750 mV (*versus* SHE). It is thought that esterase-mediated hydrolysis of the backbone, resulting in three bidentate catechol units, is required for iron release. This raises the midpoint potential of the chelated iron to around −350 mV, which is accessible to intracellular reductants, such as NADH (*E*_m_ ∼ −320 mV) (85, 95). Other exceptions to the scheme outlined above are known, most notably for the pyoverdines, the principle siderophores of some pseudomonads, where reductive iron release occurs in the periplasm (96).

### Extraction of iron from heme

In the case of many pathogenic bacteria, heme represents an important source of iron because it accounts for some 75% of the iron content of mammals (97). The heme acquisition pathway shows many parallels to siderophore uptake, perhaps reflecting the insolubility and potential toxicity of both heme and Fe^3+^.

In some cases, such as the Has system of *Pseudomonas aeruginosa*, heme scavenging proteins termed hemophores are secreted to the extracellular environment (98). These proteins ligate heme via the side chains of conserved His and Tyr residues (99, 100). In contrast to siderophores, they deliver the extracted heme to outer-membrane heme-binding proteins and are not themselves reimported to the cell. The outer-membrane proteins bind heme via two histidine residues and have a lower intrinsic affinity for heme than hemophores. However, formation of the hemophore/outer-membrane binding protein complex induces a conformational change in the hemophore, lowering its affinity for heme and ensuring transfer in the desired direction (101).

In other systems, such as Phu also from *P. aeruginosa*, the outer-membrane receptors acquire heme directly from host proteins (102). Whereas PhuR, the outer-membrane heme-binding protein of Phu, employs His/Tyr ligation of heme (103), it appears that His/His ligation is more common among these proteins (86). In either case, they bind heme with picomolar affinity and are able to extract it from host proteins such as hemoglobin or the hemoglobin-haptoglobin complex (86).

The outer-membrane heme-binding proteins are associated with 22–25-stranded β-barrel porins (Fig. 4*A*). These are also coupled to the cytoplasmic membrane potential by the TonB/ExbBD complex. As with siderophores, heme is shuttled to the inner membrane by periplasmic binding proteins (Fig. 4*C*) and imported to the cytoplasm by ABC transporters (Fig. 4*D*) (86). Once located in the cytoplasm, heme can be directly incorporated into bacterial proteins, but is more commonly subjected to oxidative degradation by heme oxygenases to liberate the iron (104). Heme acquisition systems are subject to negative regulation by the iron-dependent transcriptional regulators to avoid iron overload, but expression is also linked to sensing of heme availability by hemophores via extracytoplasmic function σ factors (105).

### Uptake of ferrous iron

Under acidic and/or anaerobic conditions, iron is predominantly in the soluble ferrous oxidation state. Consequently, bacteria have evolved mechanisms for the direct uptake of iron in this form. The solubility of Fe^2+^ means that active transport across the outer membrane of Gram-negative bacteria is not required, and it enters the periplasm by free diffusion through porins (106). Several systems have been demonstrated to import Fe^2+^ into the cytoplasm, including MntH (107), ZupT (108), YfeABCD (109), FutABC (110), EfeUOB (111), and Feo, but of these, only Feo appears both widespread and dedicated to the transport of Fe^2+^ (106).

Feo was first identified in *E. coli*, where the operon encodes three proteins, FeoA, FeoB, and FeoC (112). However, it seems that FeoC is limited to the γ-proteobacteria (88), and the most commonly occurring (54% of sequenced genomes) *feo* gene organization consists of only *feoAB*, whereas 11% of sequenced bacterial genomes contain *feoB* alone (106). FeoB is an ∼80-kDa membrane protein containing 7–12 transmembrane helices (106). A cytoplasmic domain located at the N terminus has been shown to bind and hydrolyze GTP (113–115), with hydrolysis thought to be activated by K^+^ (116). At present, it is unclear whether this supports active transport of the Fe^2+^ substrate or is used to signal the energy status of the cell. This GTPase domain is linked to the membrane-spanning helices by a GDP dissociation inhibitor domain (117) and switch regions thought to alter conformation upon nucleotide binding. The mechanism by which FeoB transports Fe^2+^ remains elusive but is thought to be mediated by binding of the metal to the sulfur atoms of Cys and Met residues located in the transmembrane helices (106).

Both *feoA* and *feoC* encode small (∼8-kDa) hydrophilic proteins. FeoA is a basic protein with pI at around pH 9.0, consistent with localization to the inner leaf of the cytoplasmic membrane (118). The protein displays significant homology to SH3 domains and possess the same fold (119). This has led to the suggestion that protein-protein interactions between FeoA and the GTPase domain of FeoB regulate the rate of nucleotide hydrolysis. Whereas deletion of *feoA* has been shown to result in a 60% reduction in Fe^2+^ transport (88), direct interaction between FeoA and FeoB has not yet been demonstrated. FeoC adopts the winged helix-turn-helix fold (120, 121) common in DNA-binding domains and from its structure has been predicted to be a repressor of transcription (118, 122). However, DNA-binding activity of FeoC remains to be demonstrated.

The Feo system exemplifies the complex interplay of iron and O_2_ metabolism that is likely a universal characteristic of bacteria. Under anaerobic conditions, the expression of ferric import systems decreases due to an increase of Fe^2+^-Fur. The *feo* operon is also negatively regulated by Fur, thereby preventing iron overload. However, at typical intracellular iron concentrations, the combined positive regulation of *feo* by ArcA and FNR alleviates Fur-mediated repression (15). In this way, anaerobic conditions lead to the repression of ferric iron uptake systems, whereas the expression of *feo*, the importer matched to the most likely available iron source, has been reported to increase 3-fold under anaerobic conditions (88).

### Iron uptake in Gram-positive bacteria

The iron acquisition pathways of Gram-positive bacteria show significant similarity to the Gram-negative systems described above despite the absence of an outer membrane and periplasmic space. Both siderophore-bound iron and heme are transported across the cell membrane by ABC transporters, whereas the Feo system is employed for the import of ferrous iron (87, 98). Iron is also extracted from internalized heme by heme oxygenase enzymes (123, 124).

Clearly, there is no requirement for either outer-membrane porins or periplasmic binding proteins. However, heme is unable to diffuse across the 15–80 nm of the peptidoglycan cell wall. Transport of heme across the cell wall is mediated by a series of proteins anchored at the cell surface. The Isd heme uptake pathway of *Staphylococcus aureus* is the most extensively studied of the Gram-positive systems and is thought to be representative of the general mechanism these bacteria employ for heme uptake (87). Four proteins are required for the transfer of heme across the cell wall to the IsdE/F ABC transporter complex. These are anchored to the cell surface by the sortases SrtA and SrtB (125, 126). In each of the four surface-anchored proteins, heme is bound at NEAT (near iron transporter) domains containing conserved Y*XXXX*Y domains in which the leading Tyr serves as a ligand to the heme iron (127). IsdB and IsdH extract heme from host proteins, whereas IsdA and IsdC shuttle the extracted heme to the ABC transporter complex with IsdC acting as the central conduit for transfer to IsdE/F (128). The unidirectional transfer of substrate is driven by the increasing affinity for heme of sequential NEAT domains in the shuttle pathway (129).

Tyrosine is an unusual heme ligand among heme-binding proteins in general but is prevalent among the proteins involved in bacterial heme acquisition. The hemophores and periplasmic binding proteins of the Gram-negative bacteria, in addition to those involved in transfer of heme across the cell wall in the Gram-positive case, all utilize tyrosine as a ligand, suggesting that its properties may be particularly suited to the capture and transfer of heme.

## Iron storage in bacteria

Iron acquired via the mechanisms described above initially enters the labile iron pool. The existence of an intracellular pool of iron not bound to proteins was initially postulated on thermodynamic grounds (130). Because iron-utilizing proteins typically bind the metal with *K_d_* on the order 10^−8^ to 10^−7^ m, it was argued that a population of free metal with concentration greater than this must exist to prevent dissociation. This was presumed to be composed of Fe^2+^ as a result of the reducing environment of the cytoplasm and the requirement for rapid ligand exchange, the kinetic lability of Fe^2+^ complexes being typically 10^4^ times greater than their Fe^3+^ counterparts.

Despite its critical importance in iron homeostasis, the chemical composition of the labile iron pool remains the source of considerable debate, in part due to the difficulty of defining the speciation of intracellular iron. Siderophores, amino acids, citrate, and low-molecular weight thiols have all been proposed as candidate ligands. Whole-cell Mössbauer spectroscopy provides the most direct empirical insight. The feature assigned to the labile iron pool has parameters typical of high-spin ferrous iron and is commonly interpreted as resulting from oxygen and nitrogen ligation (131). The relative affinities for Fe^2+^ and intracellular abundance of the proposed chelators makes citrate the most likely candidate of the oxygen donor ligands listed above. However, GSH (or equivalent low-molecular weight thiols such as mycothiol in the actinobacteria or bacillithiol in the firmicutes) is predicted to outcompete citrate at typical cytoplasmic concentrations and pH, leading to the counterproposal that the labile iron pool is dominated (up to 80%) by [Fe(H_2_O)_5_GSH]^2+^ or similar complexes (12). The prevalence of water in the coordination sphere of the Fe^2+^ would likely result in the high-spin electronic configuration reported by Mössbauer spectroscopy, despite the presence of a thiol ligand. Therefore, on balance, it seems likely that thiol-coordinated Fe^2+^ constitutes a major component of the labile iron pool.

Intriguingly, a very recent report suggests that polyphosphate acts as a hexadentate chelator of iron *in vivo*. Not only does this inorganic polymer act as a repressor of the Fenton reaction by saturating the coordination sphere of the metal; it has also been shown to act as an intracellular buffer of free iron (132). The extent to which this inorganic macromolecule contributes to either the labile iron pool or the long-term iron storage capacity of bacterial cells remains to be established.

The primary purpose of the labile iron pool is thought to be to ensure correct metalation of the iron proteome, which has been estimated to account for 60% of intracellular iron in cells grown on iron-replete (50 μm) liquid medium (133). However, as a result of the scarcity of iron, and despite the potentially catastrophic consequences of the Fenton reaction (Reaction 2), when the concentration of the labile iron pool exceeds this metabolic requirement, the excess is not simply excreted from the cell via efflux mechanisms. Rather, dedicated iron storage proteins belonging to the ferritin superfamily are employed to sequester the metal in a nonreactive state, which can be remobilized to satisfy cellular requirements during iron starvation. The signal pathway triggering the release of these iron stores remains to be elucidated, but it is reasonable to assume that the initial event would be depletion of the labile iron pool, leading to demetalation of iron-dependent transcriptional regulators.

Ferritins are found in all kingdoms of life (134). Most animal cells contain only 24-meric heteropolymers of ferritins (135). These are composed of H- and L-chains, which, respectively, contain and lack a catalytic site for iron oxidation, but which are isostructural and can thus co-assemble in different proportions, depending on the organism/tissue. In contrast, bacterial genomes commonly encode multiple predicted ferritins of different classes. These include prokaryotic analogues of the animal ferritins called Ftns, heme-containing 24-meric ferritins, called Bfrs, that are unique to bacteria, and mini-ferritins, which are dodecamers that have only been identified in prokaryotes. All prokaryotic ferritin subunits contain a catalytic center for the oxidation of iron and assemble into homopolymers (134).

**Figure 5.**
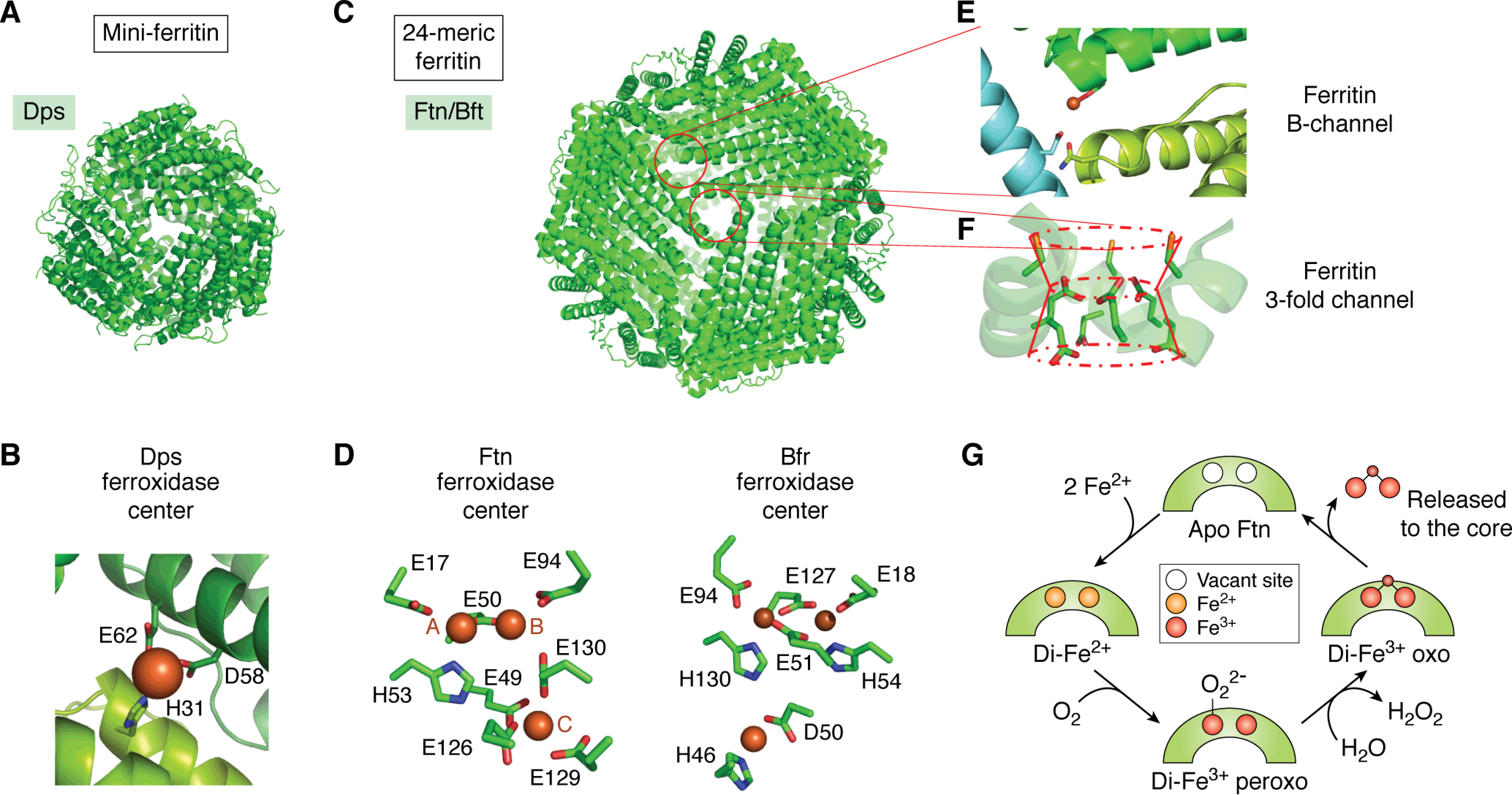
**The bacterial ferritins.** *A*, the dodecameric assembly of *L. innocua* Dps (a mini-ferritin) viewed along one of the ferritin-like 3-fold channels. *B*, single iron ion observed bound to the *L. innocua* Dps ferroxidase center. *C*, the 24-meric assembly adopted by both Ftn and Bfr viewed along the channel formed at the 3-fold symmetry axis. *D*, the ligands to iron bound at the ferroxidase center of a typical bacterial Ftn together with the associated site C (*left*) compared with the more symmetrical iron-binding environment in *E. coli* Bfr and the distinct coordination environment of the iron ion located on the inner surface of the protein (*right*). In Ftn, the higher-affinity site A has a higher coordination number than site B. *E*, expanded view of the ferritin B-channel showing Fe^2+^ bound to Asp-132 of one monomer with the potential ligands Asp-30 and Asn-63 of the two other monomers forming the channel also highlighted. *F*, side view of the ferritin 3-fold channel showing the conserved Cys (*top*), Glu (*middle*), and Asp (*bottom*) residues thought to guide the Fe^2+^ substrate toward the interior of the protein. *G*, schematic representation of the displacement mechanism that operates in some ferritins. Two equivalents of Fe^2+^ bind to the apo-ferroxidase center. Oxygen (or peroxide) binds and is reduced to peroxide (or water) by the simultaneous oxidation of both Fe^2+^ ions to Fe^3+^. Hydrolysis of the transient diferric peroxo intermediate liberates peroxide and forms a ferric-oxo precursor of the mineral core. This is displaced from the catalytic site, completing the cycle by regenerating the apo-ferroxidase center. Images produced using PDB depositions 1QGH (184) (Dps), 4ZTT (226) (Ftn), and 3E1P (161) (Bfr).

All ferritins share a four-α-helical bundle structural motif, and all except the L-chain units of animal cells contain di-iron catalytic sites, called ferroxidase centers, for the oxidation of iron (136) (Fig. 5). These are described in more detail below for each class of the bacterial proteins. Typical ferritins self-assemble into cagelike structures. The mini-ferritins form dodecamers of tetrahedral 3 3 2 symmetry with internal and external diameters of 4.5 and 9 nm, respectively (Fig. 5*A*), and possess additional helical elements at the N terminus and 2-fold axis (137). All other cage-forming ferritins possess only a short fifth helix (E) at the C terminus, altering the packing geometry. As a result, they assemble into larger rhombic dodecahedral cages possessing octahedral 4 3 2 symmetry with internal and external diameters of 8 and 12 nm, respectively (138) (Fig. 5*C*). All of these cagelike structures are permeated by channels at the vertices of their packing motifs that span the protein coat, connecting the interior cavity to bulk solution. The 4-fold channels of 24-meric ferritins are lined by the E helices, but variants in which this helix is missing are still competent to form assemblies with iron-storing capability. Rather, assembly of the protein is impaired by the disruption of residues at the C terminus of helix D (139).

The channels located at the 3-fold axes have been demonstrated to constitute the route of iron entry into animal ferritins (140). Comparatively little work has been reported on iron entry into the proteins from prokaryotes. Whereas some may also utilize the 3-fold channel (141), the so-called B-channels are used in at least a subset (142). These channels, which are found almost exclusively in prokaryotic ferritins, are formed at the 2-fold axis at the intersection between three monomeric units.

The proposal of dedicated routes for the transportation of Fe^2+^ from bulk solution through the protein coat to the site of oxidation has faced resistance due to the existence of a channel directly linking the ferroxidase center to bulk solution (143). However, there is increasing evidence that networks of carboxylate residues with conformational flexibility play key roles in Fe^2+^ transfer in all cage-forming ferritins (141, 142, 144–146). All ferritins sequester Fe^2+^ from solution and utilize an electron-accepting co-substrate, such as O_2_ or H_2_O_2_, to drive its oxidation to the Fe^3+^ state. This oxidized product is then translocated to the interior cavity, where it is stored as a hydrated ferric oxy mineral similar to ferrihydrite. Up to several thousand iron atoms per protein can be stored in this way. However, the molecular architecture of the catalytic centers carrying out this chemistry and the mechanistic detail of how it is achieved vary between the different classes of bacterial protein (147).

Expression of the mini-ferritins is usually regulated by σ factors under nutritional stress or in response to oxidative stress (148), whereas that of the 24-meric examples is usually controlled by iron-responsive transcriptional regulators. However, unlike systems for iron uptake, this cannot be achieved by a mechanism of direct repression under high concentrations of free iron. For example, under low-iron conditions in *E. coli*, production of Ftn and Bfr proteins is repressed by the small RNA RyhB (34), which binds to *ftn* and *bfr* mRNAs (as well as many others), affecting translation through a number of mechanisms that include inhibiting translation and promoting mRNA degradation. RyhB is repressed by Fur so that, at elevated iron concentrations, the metalated protein down-regulates RyhB, leading to increased levels of Ftn and Bfr proteins. It has also been reported that expression of *ftnA* can be induced by Fur in a RhyB-independent manner (35). The mRNA-binding global regulator CsrA plays an important role in iron homeostasis, through its repression of genes such as *bfr* and *dps* (149), expression of which are not required under exponential, minimal stress conditions. In *rhizobiales*, *bfr* expression is directly repressed by Irr under iron limitation, with RirA implicated in derepression as iron availability increased (72). However, in some examples of cyanobacteria, iron storage is not positively regulated by increasing iron concentration (150, 151). These observations further illustrate the complexity of cellular iron regulation.

### Iron oxidation in Ftns

The Ftns are the closest analogues to the eukaryotic ferritins found in bacteria and are also widely distributed among archaea. The crystal structures of several examples are available, including that of the most intensively studied, FtnA of *E. coli* (138). These reveal an asymmetric di-iron ferroxidase center with similar architecture to that of the H-chain ferritins from animals (Fig. 5*D*). The predicted high-affinity site (site A) is coordinated by a bidentate Glu (17 in *E. coli* FtnA numbering), His-53, and bridging Glu-50 that also coordinates the predicted lower-affinity site (site B). Coordination of the second site is completed by monodentate Glu-94 and, in most examples, a second Glu (Glu-130 in *E. coli*). This residue also ligates a third metal-binding site (site C) whose coordination is completed by a further three monodentate Glu residues (Glu-49, -126, and -129). A conserved Tyr residue (Tyr-24) is also located close to site B and forms a hydrogen bond to one of the site B ligands (Glu-94).

*In vitro* studies of recombinantly expressed proteins have been employed to interrogate the mechanism by which Ftns lay down a mineral core within their interior cavity and have revealed marked similarity to that of their counterparts from eukaryotes. Under aerobic conditions, and in the absence of alternative co-substrate such as H_2_O_2_, O_2_ binds to the freshly occupied di-Fe^2+^ center, resulting in the rapid formation of a di-Fe^3+^-peroxo intermediate that is detectable via a transient absorbance feature in the wavelength range 600–650 nm (152). Hydrolysis of this intermediate results in the formation of a ferric-oxo species thought to be the precursor of the mineral core, which is not stably bound at the ferroxidase center (153). It remains to be demonstrated how the oxidized product is transported from the site of oxidation to the cavity, although this may involve the growth of iron-oxo clusters from carboxylate side chains located on the inner surface of the protein coat in close proximity to the ferroxidase centers.

The effect of substitutions of site C residues suggests that it is involved in ferroxidase center activity in some instances, although the roles of both site C and the conserved nearby tyrosine residue appear variable between different proteins (147). Some examples of Ftn exhibit a stoichiometry of their iron/oxygen chemistry that is greater than 2:1 and is affected, together with the rate of iron oxidation, by disruption of site C, suggesting a role for this site in Fe^2+^ oxidation/catalytic turnover. In others, the site appears to function to regulate the rate of flux of the oxidized product out of the ferroxidase center, such that flux is greater in the absence of site C. A role has been postulated for the conserved Tyr as a “molecular capacitor” providing, together with the three Fe^2+^ ions bound at sites A–C, four reducing equivalents enabling the direct reduction of O_2_ to H_2_O (154). However, whereas every reported example of an H-chain like ferritin contains a Tyr residue at the equivalent position to Tyr-24 of *E. coli* FtnA, the effect of substitution of this residue (*e.g.* by Phe) is variable (147), suggesting that its function is variable. Furthermore, whereas some data support a role for conserved ferritin Tyr residues as electron donors, this is not always the case. In some instances, observation of di-Fe^3+^ peroxo species requires that assays be performed with a large excess of Fe^2+^ over ferroxidase center sites (155). These are precisely the conditions under which site C would be expected to be occupied and involvement of a third Fe^2+^ ion and oxidation of a Tyr residue would result in the direct formation of H_2_O. However, the observation of a di-Fe^3+^ peroxo species that decays to form the di-Fe^3+^ center and H_2_O_2_ indicates that H_2_O is not formed and, therefore, that the conserved Tyr does not function as a reductant.

Regardless of the route of iron exit from Ftn ferroxidase centers, it is apparent that oxidized iron is translocated from here into the interior of the protein, regenerating empty binding sites, facilitating catalytic turnover. Furthermore, the rate of this flux is increased by further incoming Fe^2+^ substrate. This “displacement” model of core formation is directly analogous to that proposed for eukaryotic ferritins (153), although the effect of helix E deletion on the ability to generate a mineral core is different between the two classes of protein (139, 156, 157), which may reflect different routes of Fe^3+^ exit from the catalytic centers.

### Iron oxidation in Bfrs

The most striking difference between the Ftns and Bfrs is the presence in the latter of 12 heme groups, located at the monomer-monomer interface of each of the subunit dimers that make up the 12 faces of the rhombic dodecahedral protein assembly. *In vitro* data indicate that the presence or absence of these prosthetic groups has little effect on the rate of iron uptake by the protein (158), particularly at low iron loadings. Instead, they are thought to promote the reductive mobilization of the mineral core (159) via their interaction with a small [2Fe-2S] cluster–containing ferredoxin, called Bfd (160), that is differentially expressed from *bfr* despite its adjacent location on many bacterial genomes.

The coordination of iron at the ferroxidase center also differs significantly between Bfr and Ftn. The catalytic center of the former is almost symmetric (Fig. 5*D*), with each metal ion coordinated by two bridging Glu residues (51 and 127, *E. coli* protein residue numbering), a His (54 at site A and 130 at site B), and a monodentate Glu (18 at site A and 94 at site B) (161). The *E. coli* protein remains the most extensively characterized example of Bfr, and, here at least, the difference in iron coordination at the ferroxidase center relative to other ferritins has an impact on the mechanism (although this is not the case for all; see below). Rather than releasing oxidized iron from the ferroxidase center into the interior of the protein, iron bound here appears to be a stable cofactor regardless of oxidation state (162), presumably as a consequence of the increased coordination number. Nevertheless, *in vitro* assays of iron mineralization activity demonstrate that the protein is able to lay down a mineral core containing up to 2800 eq of iron (163). Therefore, oxidized iron must be deposited in the interior of the protein via a route other than the displacement mechanism employed by the Ftns and other ferritins.

Crystallographic studies identified an iron-binding site, Fe_IS_, located on the inner surface of the protein that is important for function (161). This, together with a network of aromatic residues, including the tyrosine conserved in other classes of ferritin (Tyr-25 in this instance), deliver electrons into the ferroxidase center, generating Fe^3+^ within the protein cage in the process (164, 165). The reduced ferroxidase center then reacts with a further oxidizing equivalent completing the catalytic cycle. Unlike the Ftns, the stoichiometry of the Fe/O_2_ reaction is 4:1, consistent with H_2_O_2_ being a far more effective co-substrate than O_2_ for Bfr (166).

To a first approximation, the ligation of iron at the ferroxidase center of *Pseudomonas aeruginosa* Bfr (BfrB)^4^ is identical to that in the *E. coli* protein. However, the structure of the protein derived from crystals subjected to different soaking conditions demonstrated conformational flexibility in residue His-130 (167). Whereas this residue acts as a ligand to iron in site B for structures in which the ferroxidase center is occupied, these sites are vacant in crystals formed from the protein as isolated, and His-130 in these structures is rotated relative to those with metal-containing active sites such that it would be unable to bond to a metal ion located at site B. These observations led to the proposal that the ferroxidase center of *P. aeruginosa* Bfr behaves as a gated pore for iron entry to the protein and a displacement mechanism of core formation akin to that of the Ftns. It is noteworthy that the rate at which the *P. aeruginosa* and *E. coli* Bfr proteins oxidize Fe^2+^ following binding of the metal to apo-ferroxidase centers is similar, but the former is able to lay down a mineral core at a rate far greater than the latter, consistent with mechanistic differences between them. The structure-function relationships governing these differences have not yet been resolved.

### The roles of Ftn and Bfr vary between organisms

In *E. coli*, an *ftnA* deletion mutant exhibited marked impairment of growth compared with the WT strain on transfer from iron-replete to iron-deficient conditions (168). This phenotype was not observed for the *bfr* mutant, suggesting a role other than iron storage for this protein, possibly in oxidative stress response. In contrast, deletion of the *bfrB* gene in *P. aeruginosa* severely impairs the ability of the organism to accumulate iron as FtnA does not sequester a mineral core even in the absence of Bfr. Deletion of *bfd* or disruption of the Bfr/Bfd interaction elicits an iron starvation response, even under iron-replete conditions due to irreversible deposition of iron within the BfrB core (169). Therefore, it appears that the roles of Ftn and Bfr are reversed in the two organisms, and this may correlate with the reported differences in mineralization mechanism. A similarly variable picture is emerging from studies of ferritins in other organisms. For example, in *Salmonella enterica*, Bfr appears to be the major iron store (170), whereas, in *M. tuberculosis*, Ftn (previously known as BfrB^4^) is important for virulence (171) and under high iron levels, whereas Bfr (BfrA) appears to be important for recycling iron under low iron levels (172). In the strictly anaerobic sulfate-reducing bacterium *Desulfovibrio vulgaris*, Bfr plays an important role in protecting the organism from O_2_, which is normally toxic to such bacteria (173).

### Iron oxidation by Dps/Dpr proteins

The Dps (DNA-binding proteins under starvation) proteins are composed of 12 identical α-helical subunits (rather than 24) and are consequently also known as mini-ferritins. They are significantly up-regulated during stationary phase or periods of oxidative stress (174). In addition to consuming the Fenton reagents Fe^2+^ and H_2_O_2_, they bind nonspecifically to DNA (175, 176). This provides a physical barrier and can induce a crystalline transition in the nucleoid (177, 178), both of which are thought to protect against oxidative damage. The affinity of these proteins for DNA is thought to be due to a “tail” at the N terminus of the peptide that is rich in positively charged residues providing a favorable electrostatic interaction (179–181). Dps proteins protect against multiple stress factors but require both DNA-binding and ferroxidase activity in all cases (181). We note that homologues of Dps proteins have been identified in nutritionally deficient stationary phase cultures that exhibit antioxidant activity but do not bind to DNA. These proteins, termed Dpr, are under the control of transcriptional regulators that respond to redox status/oxidative stress (*e.g.* PerR in *Streptococcus pyogenes* (182) or RitR in *Streptococcus pneumoniae* (183)).

The subunit arrangement of Dps 12-mer mini-ferritins results in a change in the symmetry of the channels penetrating the protein coat (2-fold channels and two classes of 3-fold channel) compared with the 24-mer proteins. One of the classes of 3-fold channel is unique to these proteins, whereas the second is similar to the 3-fold channels of other ferritins and is thought to constitute the route of iron entry (179, 184). The location and structure of the ferroxidase center is also unique among the cage-forming ferritins. Rather than being buried within the four-α-helical bundle, it is located at the interface between the two protomers of each subunit dimer. In the majority of structural models derived from diffraction data, this site contains only a single ion coordinated by conserved carboxylate and histidine residues (184–186). The first reported example was from the Dps of *Listeria innocua*, with iron ions coordinated by Glu-62 and Asp-58 of one protomer and His-31 of its partner within the subunit dimer (184) (Fig. 5*B*).

A di-iron form of the catalytic site, modeled by placing an iron ion at the position of a nearby ordered water, suggested that Glu-62 might bridge the two metals, with His-43 from the same protomer as His-31 being the only other potential ligand. In the few cases where two metal ions have been observed at the ferroxidase center, the second metal has a significantly larger temperature factor than its surroundings, indicating significant lability of this site (187). Attempts to assess iron binding by fluorescence quenching indicated 24 eq of iron per protein upon the addition of Fe^3+^ but only 12 eq when titrating with Fe^2+^ (188). This has led to the proposal that the di-iron site is only formed as an intermediate in the oxidation reaction of Dps, in contrast to the 24-mer cages, where the occupancy of both sites is thought to be a prerequisite for rapid reactivity with either O_2_ or H_2_O_2_. Consistent with a role in combating oxidative stress, the Dps centers utilize H_2_O_2_ as the co-substrate for Fe^2+^ oxidation, being significantly less reactive toward O_2_ (189).

### Iron storage in cyanobacteria

A survey of the distribution of iron storage proteins in cyanobacterial genomes revealed significant differences from other bacteria, with only around 12% of genomes containing a homolog of FtnA. A great many of the genomes of marine picocyanobacteria (*Prochlorococcus* and *Synechococcus*) contain a distinct class of ferritin that differs from the classic Ftn proteins in that the coordinating side chains that make up site C are absent in the peptide chain. An example from *Synechococcus* sp. CC9311, *Syn*Ftn, was found to be up-regulated in response to exposure to elevated concentrations of copper (88). Furthermore, several of the marine picocyanobacteria possess genes encoding homologs of both *Syn*Ftn and FtnA. Together, these observations suggest that *Syn*Ftn may have a role in oxidative or general stress response rather than iron homeostasis. *In vitro* characterization of this protein demonstrated that, whereas the mineral core is generated via the typical displacement of oxidized iron from the catalytic center, the oxidation of this site proceeds via a mixed valent Fe^2+^/Fe^3+^ intermediate not previously observed during ferritin activity (or indeed the oxidation of any other O_2_-activated di-iron protein save one), where di-Fe^2+^ sites are oxidized directly to di-Fe^3+^ peroxo species. The Fe^2+^/Fe^3+^ intermediate oxidizes to a metastable di-Fe^3+^ form in ∼10 s at atmospheric O_2_ concentration. This breaks down to release mineral product to the protein interior and regenerate apo sites able to bind further equivalents of Fe^2+^ and initiate another reaction cycle. In further contrast to other bacterial Ftns, the di-Fe^2+^ form of *Syn*Ftn ferroxidase centers is unreactive toward H_2_O_2_, utilizing only O_2_ as co-substrate (190).

Whereas the genomes of many cyanobacteria lack homologs of any of the characterized 24-mer ferritins, homologs of the mini-ferritins appear to be widespread (191), and these have been shown to have roles in iron homeostasis, in addition to oxidative stress response (192). Some genomes encode multiple examples. Among the most extensively studied are those of *Nostoc punctiforme*, a filamentous cyanobacterium in which the majority of cells in filaments are in a vegetative state and perform photosynthesis, but around 5% form heterocysts—differentiated cells that perform an N_2_-fixing function. *N. punctiforme* encodes five Dps homologs (193), annotated NpDps1–5 (194), that are differentially transcribed, depending on cell type. Of these, NpDps1–3 have been designated typical Dps-like proteins based on sequence homology (195), with NpDps2 predominantly expressed in photosynthetic vegetative cells and the others predominantly in heterocysts. As with the Dps proteins of pathogens, they also use H_2_O_2_ as the preferred oxidant. Whereas this group of proteins exhibit some degree of co-regulation, individual proteins are also thought to be up-regulated in response to a variety of environmental cues. NpDps1 is expressed in response to low temperature (196), whereas NpDps2 confers resistance to oxidative stress induced both by exogenous H_2_O_2_ (191) and high light levels (194) and is also expressed in response to heat shock. NpDps5 appears to perform a similar role to NpDps2, conferring resistance to both H_2_O_2_ (197) and light-induced oxidative stress (194), but is also involved in iron homeostasis. The ligation of the ferroxidase center in this protein differs markedly from canonical Dps proteins and closely resembles that of bacterial Bfrs discussed above (191). Finally, NpDps4 possesses unusually His-rich ligation of iron at the ferroxidase center and utilizes only O_2_ and not H_2_O_2_ as an oxidant for iron (198). Accordingly, a role for this protein has been proposed as an O_2_ scavenger within heterocysts where nitrogenase activity requires that a microoxic (<10 μm O_2_) environment be maintained (199). Based on sequence comparisons with other Dps proteins, it has been suggested that this type of reaction center, which is common among, but restricted to, the cyanobacteria (198) be classified as the His-type ferroxidase center.

### Iron storage in encapsulins

Encapsulins are large macromolecular assemblies, similar in structure to virus capsids. They are composed of proteins possessing the HK97 fold, a ubiquitous fold among proteins forming virus shells and other large compartments. (200). Two major classes of encapsulin cage architecture have been reported, distinguished by their triangulation number, *T*. The faces of the encapsulin are composed of regular hexagonal and pentagonal units, with curvature to create the enclosed 3D structure introduced by the latter. *T* defines the distance separating pentagonal units and therefore the size of the protein cage. Those with triangulation number *T* = 1 have the smallest possible enclosed volume and are composed of 60 identical subunits (201), whereas larger cages composed of 180 subunits possess a triangulation number *T* = 3 (202). Diameters range from 24 to 32 nm. Very recently, a new type of encapsulin was reported, from the bacterium *Quasibacillus thermotolerans*, which is larger still, with a diameter of 42 nm and novel *T* = 4 topology (203).

**Figure 6.**
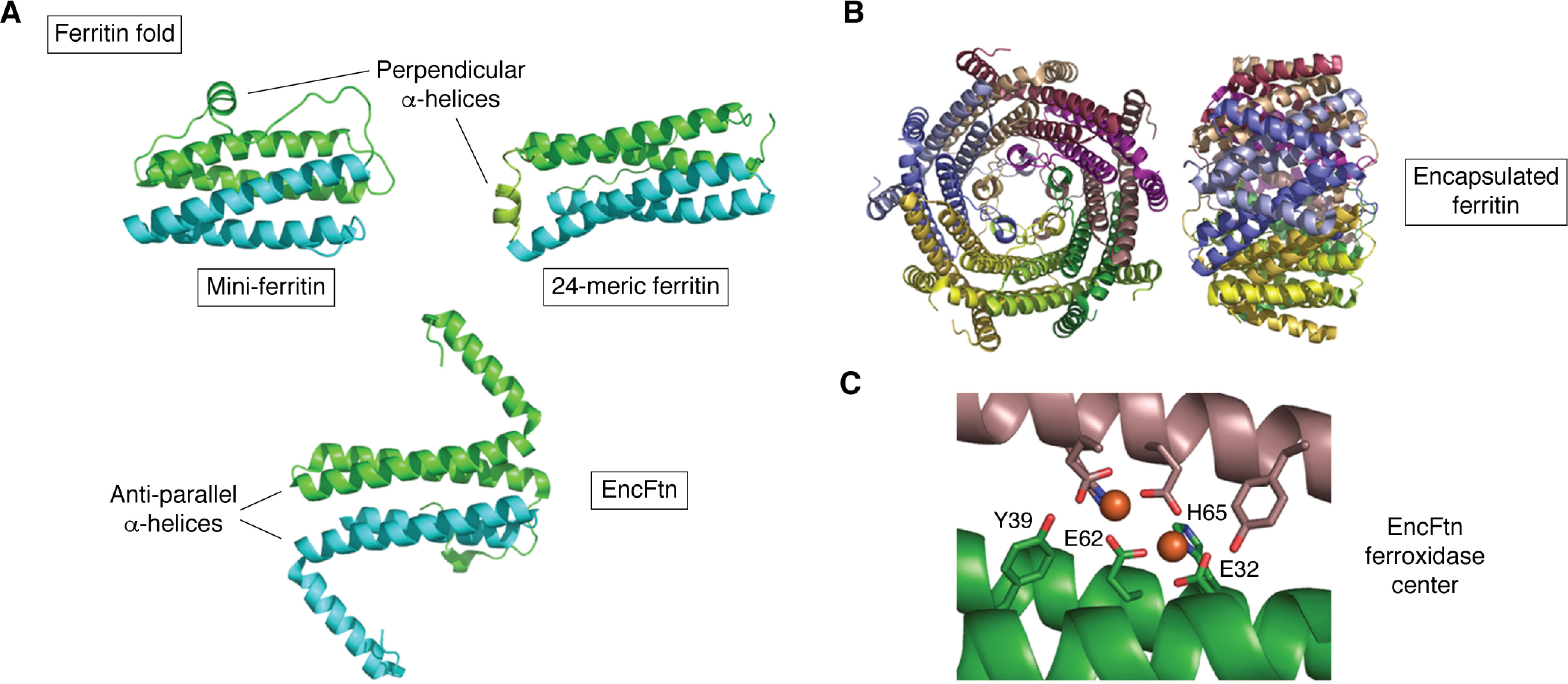
**Encapsulated ferritins.** *A*, the ferritin fold is made up of two homologous pairs of anti-parallel α-helices (136), here *colored green* and *cyan*. In the true, cage-forming ferritins, these are connected via a loop joining helices *B* and *C*. Short helices running perpendicular to the long axis of the bundle help to template cage formation in the mini-ferritins (*top*) or 24-meric examples (*middle*). Members of the superfamily that do not form cages, such as EncFtn (*bottom*), are associated with further extended secondary structure elements, such as the membrane-spanning helices of MbfA or the large additional helices of EncFtn, which prevent assembly into cages. *B*, the annular pentamer of dimers adopted by the majority of encapsulated ferritins. *C*, the ferroxidase center of a typical encapsulated ferritin highlighting the noncrystallographic 2-fold symmetry of the iron environment. For clarity, only the ligands provided by the lower of the two protomers have been labeled. Images produced using PDB deposition 5N5E (206) (EncFtn).

These large assemblies have the ability to encapsulate cargo proteins, which are targeted to the capsid by short C-terminal sequences (204). Among the cargo proteins of encapsulins are ferritin-like proteins. These encapsulated ferritins (EncFtn) are members of the ferritin superfamily that possess ferroxidase activity but do not themselves assemble to form cages (205). Whereas they are not as ubiquitous as their cage-forming counterparts, they have been identified in a wide range of bacterial and archaeal species from diverse environments (206). In all cases, these EncFtn proteins assemble into dimers; most assemble further to form annular pentamers of dimers (Fig. 6*B*). As a result, all lack the intrinsic ability to solubilize mineral cores, requiring localization within encapsulin cages to do so (207, 208). Due to their greater size, encapsulin complexes containing EncFtn are capable of storing at least 4 times (205, 207) (and in the case of the *Q. thermotolerans* encapsulin, ∼10 times) the amount of iron associated with the classical ferritins described above.

Most EncFtn proteins differ from the other members of the ferritin superfamily in that the protein monomer essentially consists of two antiparallel α-helices, with an additional shorter helix at the C terminus. The classic four-α-helical motif of the ferritins is achieved by the association of these subunits into dimers. The *Q. thermotolerans* EncFtn is distinct in that its subunit consists of a four-α-helical bundle, which assembles into dimers.

The di-iron ferroxidase center has an approximate 2-fold symmetry axis (Fig. 6*C*), with each of the two monomers contributing identical ligand sets (*cf.* the case with the Dps proteins). In *Q. thermotolerans* EncFtn, each iron is coordinated by a bridging Glu and two His residues. In most others, each monomer provides a bridging Glu such that there are two equivalent Glu residues bridging the metals. Each iron is also ligated by a His and a bidentate Glu, with the two additional ligands located on the same monomer. The hydroxyl of a Tyr residue is located 4.5 Å from each of the irons of the ferroxidase center in most structures, but their significance is not known, as the mechanism of iron oxidation at EncFtn centers remains to be elucidated (206). Whereas these proteins have been demonstrated to support the catalytic oxidation of Fe^2+^ in the presence of O_2_, and this has been shown to be inhibited by Zn^2+^, it is not known whether O_2_ or H_2_O_2_ is the preferred substrate of EncFtn.

## Efflux of iron from the cell

Because iron has long been viewed as a growth-limiting nutrient, mechanisms of iron export from bacterial cells are a relatively underresearched area. However, it is apparent that under certain circumstances, simply down-regulating iron acquisition may not be sufficient to ensure cellular survival. Chief among these is ROS assault, which arises from the close link between oxidative stress and elevated levels of intracellular iron mediated by the Fenton reaction. In some cases at least, countering this assault necessitates the active removal of iron from the cell, but the discovery of the efflux systems responsible is a relatively recent development (209). Consequently, the understanding of these systems lacks the mechanistic detail available for the molecules of iron sensing, import, and storage. However, the main features of the four known classes of bacterial iron efflux systems are outlined below.

P-type ATPases are cytoplasmic membrane proteins that consist of a transmembrane domain containing 6–8 helices, an ATP-binding domain, and a soluble actuator domain. Examples with iron-exporting activity belong to the P_1B4_ family and have been identified in *Bacillus subtilis* (PfeT) (210), *Listeria monocytogenes* (FrvA) (211), *M. tuberculosis* (CtpD) (212), the group A *Streptococci* (PmtA) (213, 214), and *Sinorhizobium meliloti* (Nia) (215). Where the regulator of transcription has been identified, it is Fur and/or PerR, indicating the dual role in iron-mediated and peroxide stress response.

Cation diffusion facilitator (CDF) metal ion transporters are ubiquitous among prokaryotes and eukaryotes, with a wide range of cations transported. The proteins consist of six transmembrane helices with a histidine-rich loop interconnecting transmembrane helices 4 and 5. A soluble cytoplasmic domain is located at the C terminus. Little is known about the factors influencing metal ion selectivity, but iron-exporting activity has been reported for examples from *E. coli* (YiiP or FieF) (216), *P. aeruginosa* (AitP) (217), and *Shewonella oneidensis* (FeoE) (218). Unlike the P-type ATPase systems, the transcriptional regulators of their expression have yet to be identified.

Major facilitator superfamily proteins function in the transmembrane transport of cations, but the mechanism by which they achieve this is not well-understood. They are made up of two domains, each consisting of six transmembrane helices. IceT of *Salmonella typhimurium* (219) is the only reported example with iron-exporting activity and is under the transcriptional control of the BaeSR system that regulates antibiotic resistance and efflux.

Membrane-bound ferritins do not form cages and are therefore are not *bona fide* ferritins (Fig. 6*A*). However, they contain a ferritin-like domain at the N terminus that has ferroxidase activity (220). Located on the cytoplasmic side of the membrane, this domain is required for iron transport. The C-terminal domain is membrane-spanning and has significant sequence homology to the vacuolar iron transporters, such as VIT1 of *Arabidopsis thaliana*. Reported examples are found in the α-proteobacteria *A. tumefaciens* (221) and *B. japonicum* (220), where they are thought to be important in oxidative stress response during the infection of plants. Annotated as MbfA, their transcription is under the control of Irr.

## Concluding remarks

**Figure 7.**
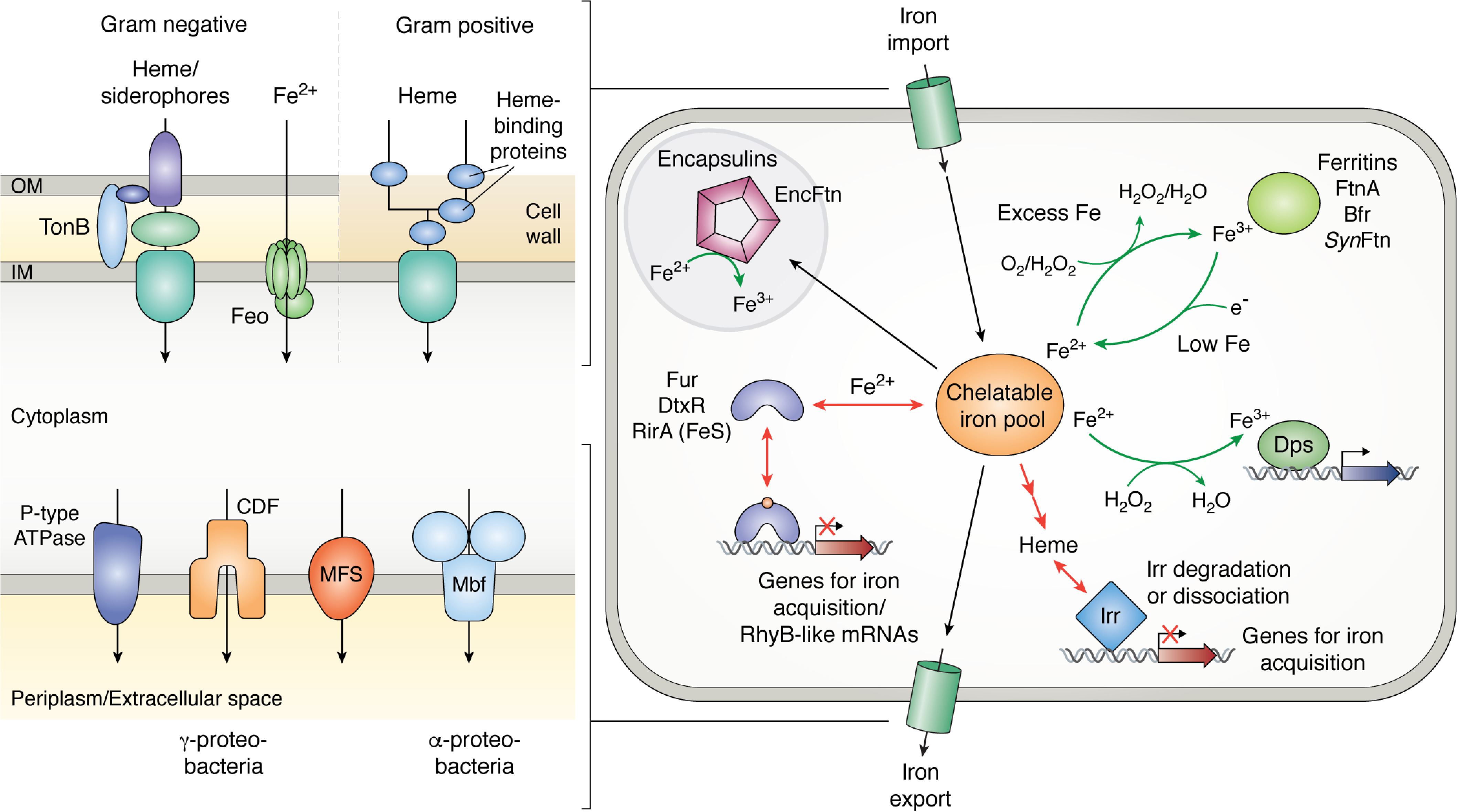
**Schematic overview of the major components of iron sensing and detoxification found in bacterial cells.** Note that not all of these components are present in a single bacterial cell. Regulatory proteins are shown here as repressors but, in some cases, can also act as activators. Encapsulins are large protein compartments that house EncFtn ferritin-like proteins. The fate of iron stored in encapsulins and in Dps proteins is not clear, although it is likely that at some point, it becomes bioavailable again. Ftn, Bfr, and Dps do not appear to be distributed according to phyla. Fur is the transcriptional regulator in most bacteria but is replaced by DtxR/IdeR in some actinobacteria. In the α-proteobacteria, Fur plays a diminished role in iron homeostasis, with the majority of these functions being performed by Irr. In some *rhizobiales*, this is achieved in conjunction with a second global regulator, RirA. Import of siderophores and heme across the cytoplasmic membrane (*IM*) is performed by ABC transporters in all known cases, and Feo is the major importer of Fe^2+^. In Gram-negative bacteria, heme and siderophores are imported to the periplasm by outer-membrane (*OM*) porins, whereas a network of heme-binding proteins transports this cofactor across the cell wall of the Gram-positive bacteria. Characterized Fe^2+^ export systems are rare, but P-type ATPases are the most widely distributed. IceT of *S. typhimurium* is the only example of the MFS characterized to date, whereas the CDF proteins are limited to γ-proteobacteria and the MbfA proteins to α-proteobacteria. YiiP from *E. coli* is the only Fe^2+^ efflux pump for which the structure has been solved (227).

In this review, we have attempted to provide an overview of the current understanding of iron detoxification by bacteria, as summarized in Fig. 7. The modes of operation of the Fe^2+^-binding transcriptional regulators Fur and DtxR are now understood in molecular detail, and a great many genes under their control have been identified. Work is now under way unraveling the complex interplay between these and other regulators involved in response to oxidative and nutritional stress, and a great deal of progress is being made in this area. Whereas no crystal structures are yet available for the iron-responsive transcriptional regulators of the α-proteobacteria, Irr and RirA, the mechanism by which they use iron-containing prosthetic groups to sense the concentration of the metal has been established, as has the molecular basis of their ability to also sense O_2_. Also, an understanding of the interplay between these two regulators and the genes that they control is emerging. The common thread between all is a down-regulation of iron acquisition pathways and up-regulation of iron storage systems in response to elevated iron concentrations (Fig. 7).

The greater number of ferritins encoded in bacterial genomes compared with those of animals possibly reflects the greater need for bacterial cells to respond to a variety of environmental stresses that are linked to iron, from iron deprivation to ROS- and RNS-induced oxidative stress. Reported growth inhibition of deletion mutants compared with WT strains of various bacteria consistently supports the notion that ferritin minerals are viable stores of nutritionally available iron.

A recent study of *E. coli* revealed that exponentially growing cells contain a significant proportion of iron in the reduced state, with ferric mineral iron only accumulating in stationary phase (131). This fascinating result highlights the importance of precise physiological conditions in determining the extent to which the quota of iron within *E. coli* cells is oxidized to the ferric state. It suggests that the redox state of intracellular iron in bacterial cells is a more subtle balance of the oxidoreductase activity of ferritins and the reducing environment created by low-molecular weight thiols than has previously been appreciated. These observations were rationalized in terms of an expansion of the “respiratory shield” hypothesis originally proposed for mitochondria. In essence, diffusion of O_2_ across either the mitochondrial or, in this instance, the cytoplasmic membrane is prevented by its consumption during respiration. Thus, the enzymes of the respiratory chain form a shield, creating a microaerobic environment in the interior matrix/cytoplasm that protects O_2_-sensitive proteins and cofactors from damage during normal respiratory function. The static dissolved O_2_ concentration inside mitochondria has been estimated at around 1 μm (131), and that in the cytoplasm of bacterial cells is assumed to be similar during exponential growth. This emphasizes an important difference between the environments in which the ferritins of bacteria and animals operate. Respiration in animal cells is restricted to mitochondria, and ferritins located in the cytosol are therefore exposed to a significantly greater O_2_ concentration than their bacterial counterparts for which peroxide would logically be expected to be an available co-substrate for iron oxidation.

A topical debate in the field of ferritin research is the existence or otherwise of a 'universal' mechanism of iron oxidation. This was proposed based on similarities between different ferritins in terms of their mineralized iron products, their iron-binding stoichiometries, and common intermediates that are formed during Fe^2+^ oxidation/mineralization (222). The above considerations would argue for variation between bacterial and animal ferritins based on availability of potential substrates. Furthermore, the existence of multiple well-described mechanisms, including the very recent discovery of extremely unusual iron-O_2_ chemistry in the cyanobacterial ferritin *Syn*Ftn (190), which share only the broadest characteristics, provides ample evidence that such variation exists even within bacterial ferritins. Nature never fails to impress with the different ways in which it has found solutions to similar, if not identical, problems. The encapsulated ferritins provide the most recently discovered and a particularly striking example of the variety of solutions to the problems posed by iron.

## Footnotes

4 BfrB is in fact the only Bfr found in *P. aeruginosa*; the protein originally named BfrA was subsequently found to be of the Ftn class.

## References

[1] Beinert H., Holm R. H., and Münck E. (1997) Iron-sulfur clusters: nature's modular, multipurpose structures. Science 277, 653–659 10.1126/science.277.5326.653 9235882

[2] Gamba I., Codolà Z., Lloret-Fillol J., and Costas M. (2017) Making and breaking of the O-O bond at iron complexes. Coord. Chem. Rev. 334, 2–24 10.1016/j.ccr.2016.11.007

[3] Berks B. C., Ferguson S. J., Moir J. W. B., and Richardson D. J. (1995) Enzymes and associated electron transport systems that catalyse the respiratory reduction of nitrogen oxides and oxyanions. Biochim. Biophys. Acta 1232, 97–173 10.1016/0005-2728(95)00092-5 8534676

[4] Kappler U., and Maher M. J. (2013) The bacterial SoxAX cytochromes. Cell. Mol. Life Sci. 70, 977–992 10.1007/s00018-012-1098-y 22907414 PMC11113948

[5] Poulos T. L. (2014) Heme enzyme structure and function. Chem. Rev. 114, 3919–3962 10.1021/cr400415k 24400737 PMC3981943

[6] Zhang Y. F., Sen S., and Giedroc D. P. (2020) Iron acquisition by bacterial pathogens: beyond Tris-catecholate complexes. Chembiochem 21, 1955–1967 10.1002/cbic.201900778 32180318 PMC7367709

[7] Touati D. (2000) Iron and oxidative stress in bacteria. Arch. Biochem. Biophys. 373, 1–6 10.1006/abbi.1999.1518 10620317

[8] Imlay J. A. (2013) The molecular mechanisms and physiological consequences of oxidative stress: lessons from a model bacterium. Nat. Rev. Microbiol. 11, 443–454 10.1038/nrmicro3032 23712352 PMC4018742

[9] Reniere M. L. (2018) Reduce, induce, thrive: bacterial redox sensing during pathogenesis. J. Bacteriol. 200, e00128–18 10.1128/jb.00128-18 29891640 PMC6088161

[10] Chandrangsu P., Rensing C., and Helmann J. D. (2017) Metal homeostasis and resistance in bacteria. Nat. Rev. Microbiol. 15, 338–350 10.1038/nrmicro.2017.15 28344348 PMC5963929

[11] Chandrangsu P., Loi V. V., Antelmann H., and Helmann J. D. (2018) The role of bacillithiol in Gram-positive firmicutes. Antioxid. Redox Signal. 28, 445–462 10.1089/ars.2017.7057 28301954 PMC5790435

[12] Hider R. C., and Kong X. L. (2011) Glutathione: a key component of the cytoplasmic labile iron pool. Biometals 24, 1179–1187 10.1007/s10534-011-9476-8 21769609

[13] Keyer K., and Imlay J. A. (1996) Superoxide accelerates DNA damage by elevating free-iron levels. Proc. Natl. Acad. Sci. U. S. A. 93, 13635–13640 10.1073/pnas.93.24.13635 8942986 PMC19375

[14] Jacques J. F., Jang S., Prévost K., Desnoyers G., Desmarais M., Imlay J., and Massé E. (2006) RyhB small RNA modulates the free intracellular iron pool and is essential for normal growth during iron limitation in *Escherichia coli*. Mol. Microbiol. 62, 1181–1190 10.1111/j.1365-2958.2006.05439.x 17078818

[15] Beauchene N. A., Mettert E. L., Moore L. J., Keleş S., Willey E. R., and Kiley P. J. (2017) O_2_ availability impacts iron homeostasis in *Escherichia coli*. Proc. Natl. Acad. Sci. U. S. A. 114, 12261–12266 10.1073/pnas.1707189114 29087312 PMC5699043

[16] Koh E. I., Robinson A. E., Bandara N., Rogers B. E., and Henderson J. P. (2017) Copper import in *Escherichia coli* by the yersiniabactin metallophore system. Nat. Chem. Biol. 13, 1016–1021 10.1038/nchembio.2441 28759019 PMC5562518

[17] Schäffer S., Hantke K., and Braun V. (1985) Nucleotide-sequence of the iron reglulatory gene *fur*. Mol. Gen. Genet. 200, 110–113 10.1007/BF00383321 2993806

[18] Baichoo N., and Helmann J. D. (2002) Recognition of DNA by Fur: a reinterpretation of the Fur box consensus sequence. J. Bacteriol. 184, 5826–5832 10.1128/jb.184.21.5826-5832.2002 12374814 PMC135393

[19] Escolar L., de Lorenzo V., and Pérez-Martín J. (1997) Metalloregulation *in vitro* of the aerobactin promoter of *Escherichia coli* by the Fur (ferric uptake regulation) protein. Mol. Microbiol. 26, 799–808 10.1046/j.1365-2958.1997.6211987.x 9427409

[20] Pecqueur L., D'Autreaux B., Dupuy J., Nicolet Y., Jacquamet L., Brutscher B., Michaud-Soret I., and Bersch B. (2006) Structural changes of *Escherichia coli* ferric uptake regulator during metal-dependent dimerization and activation explored by NMR and x-ray crystallography. J. Biol. Chem. 281, 21286–21295 10.1074/jbc.M601278200 16690618

[21] Pérard J., Covès J., Castellan M., Solard C., Savard M., Miras R., Galop S., Signor L., Crouzy S., Michaud-Soret I., and de Rosny E. (2016) Quaternary structure of Fur proteins, a new subfamily of tetrameric proteins. Biochemistry 55, 1503–1515 10.1021/acs.biochem.5b01061 26886069

[22] Pohl E., Haller J. C., Mijovilovich A., Meyer-Klaucke W., Garman E., and Vasil M. L. (2003) Architecture of a protein central to iron homeostasis: crystal structure and spectroscopic analysis of the ferric uptake regulator. Mol. Microbiol. 47, 903–915 10.1046/j.1365-2958.2003.03337.x 12581348

[23] Fuangthong M., and Helmann J. D. (2003) Recognition of DNA by three ferric uptake regulator (Fur) homologs in *Bacillus subtilis*. J. Bacteriol. 185, 6348–6357 10.1128/jb.185.21.6348-6357.2003 14563870 PMC219410

[24] Sarvan S., Charih F., Askoura M., Butcher J., Brunzelle J. S., Stintzi A., and Couture J. F. (2018) Functional insights into the interplay between DNA interaction and metal coordination in ferric uptake regulators. Sci. Rep. 8, 7410 10.1038/s41598-018-25157-6 29739988 PMC5940780

[25] Sarvan S., Butcher J., Stintzi A., and Couture J. F. (2018) Variation on a theme: investigating the structural repertoires used by ferric uptake regulators to control gene expression. Biometals 31, 681–7044 10.1007/s10534-018-0120-8 30014354

[26] Mills S. A., and Marletta M. A. (2005) Metal binding characteristics and role of iron oxidation in the ferric uptake regulator from *Escherichia coli*. Biochemistry 44, 13553–13559 10.1021/bi0507579 16216078

[27] Deng Z. Q., Wang Q., Liu Z., Zhang M. F., Machado A. C. D., Chiu T. P., Feng C., Zhang Q., Yu L., Qi L., Zheng J. G., Wang X., Huo X. M., Qi X. X., Li X. R., et al (2015) Mechanistic insights into metal ion activation and operator recognition by the ferric uptake regulator. Nat. Commun. 6, 7642 10.1038/ncomms8642 26134419 PMC4506495

[28] Choi J., and Ryu S. (2019) Regulation of iron uptake by fine-tuning the iron responsiveness of the iron sensor Fur. Appl. Environ. Microbiol. 85, e03026–18 10.1128/aem.03026-18 30824449 PMC6495754

[29] Delany I., Rappuoli R., and Scarlato V. (2004) Fur functions as an activator and as a repressor of putative virulence genes in *Neisseria meningitidis*. Mol. Microbiol. 52, 1081–1090 10.1111/j.1365-2958.2004.04030.x 15130126

[30] Seo S. W., Kim D., Latif H., O'Brien E. J., Szubin R., and Palsson B. O. (2014) Deciphering Fur transcriptional regulatory network highlights its complex role beyond iron metabolism in *Escherichia coli*. Nat. Commun. 5, 4910 10.1038/ncomms5910 25222563 PMC4167408

[31] Yu C. X., and Genco C. A. (2012) Fur-mediated activation of gene transcription in the human pathogen *Neisseria gonorrhoeae*. J. Bacteriol. 194, 1730–1742 10.1128/JB.06176-11 22287521 PMC3302472

[32] Pinochet-Barros A., and Helmann J. D. (2020) *Bacillus subtilis* Fur is a transcriptional activator for the PerR-repressed *pfeT* gene, encoding an iron efflux pump. J. Bacteriol. 202, e00697–19 10.1128/jb.00697-19 31988078 PMC7099144

[33] McHugh J. P., Rodríguez-Quiñones F., Abdul-Tehrani H., Svistunenko D. A., Poole R. K., Cooper C. E., and Andrews S. C. (2003) Global iron-dependent gene regulation in *Escherichia coli*—a new mechanism for iron homeostasis. J. Biol. Chem. 278, 29478–29486 10.1074/jbc.M303381200 12746439

[34] Massé E., and Gottesman S. (2002) A small RNA regulates the expression of genes involved in iron metabolism in *Escherichia coli*. Proc. Natl. Acad. Sci. U. S. A. 99, 4620–4625 10.1073/pnas.032066599 11917098 PMC123697

[35] Nandal A., Huggins C. C. O., Woodhall M. R., McHugh J., Rodríguez-Quiñones F., Quail M. A., Guest J. R., and Andrews S. C. (2010) Induction of the ferritin gene (*ftnA*) of *Escherichia coli* by Fe^2+^-Fur is mediated by reversal of H-NS silencing and is RyhB independent. Mol. Microbiol. 75, 637–657 10.1111/j.1365-2958.2009.06977.x 20015147

[36] Isabella V., Wright L. F., Barth K., Spence J. M., Grogan S., Genco C. A., and Clark V. L. (2008) *cis*- and *trans*-acting elements involved in regulation of *norB* (*norZ*), the gene encoding nitric oxide reductase in *Neisseria gonorrhoeae*. Microbiology 154, 226–239 10.1099/mic.0.2007/010470-0 18174141

[37] Butcher J., Sarvan S., Brunzelle J. S., Couture J. F., and Stintzi A. (2012) Structure and regulon of *Campylobacter jejuni* ferric uptake regulator Fur define apo-Fur regulation. Proc. Natl. Acad. Sci. U. S. A. 109, 10047–10052 10.1073/pnas.1118321109 22665794 PMC3382491

[38] Carpenter B. M., Gilbreath J. J., Pich O. Q., McKelvey A. M., Maynard E. L., Li Z. Z., and Merrell D. S. (2013) Identification and characterization of novel *Helicobacter pylori* apo-Fur-regulated target genes. J. Bacteriol. 195, 5526–5539 10.1128/JB.01026-13 24097951 PMC3889615

[39] Davies B. W., Bogard R. W., and Mekalanos J. J. (2011) Mapping the regulon of *Vibrio cholerae* ferric uptake regulator expands its known network of gene regulation. Proc. Natl. Acad. Sci. U. S. A. 108, 12467–12472 10.1073/pnas.1107894108 21750152 PMC3145737

[40] Embree M., Qiu Y., Shieu W. D., Nagarajan H., O'Neil R., Lovley D., and Zengler K. (2014) The iron stimulon and Fur regulon of *Geobacter sulfurreducens* and their role in energy metabolism. Appl. Environ. Microbiol. 80, 2918–2927 10.1128/AEM.03916-13 24584254 PMC3993298

[41] Neto J. F. D., Lourenço R. F., and Marques M. V. (2013) Global transcriptional response of *Caulobacter crescentus* to iron availability. BMC Genomics 14, 549 10.1186/1471-2164-14-549 23941329 PMC3751524

[42] Díaz-Mireles E., Wexler M., Sawers G., Bellini D., Todd J. D., and Johnston A. W. B. (2004) The Fur-like protein Mur of *Rhizobium leguminosarum* is a Mn^2+^-responsive transcriptional regulator. Microbiology 150, 1447–1456 10.1099/mic.0.26961-0 15133106

[43] Fillat M. F. (2014) The Fur (ferric uptake regulator) superfamily: diversity and versatility of key transcriptional regulators. Arch. Biochem. Biophys. 546, 41–52 10.1016/j.abb.2014.01.029 24513162

[44] Touati D., Jacques M., Tardat B., Bouchard L., and Despied S. (1995) Lethal oxidative damage and mutagenesis are generated by iron in Δ*fur* mutants of *Escherichia coli*—protective role of superoxide dismutate. J. Bacteriol. 177, 2305–2314 10.1128/jb.177.9.2305-2314.1995 7730258 PMC176885

[45] Berges M., Michel A.-M., Lassek C., Nuss A. M., Beckstette M., Dersch P., Riedel K., Sievers S., Becher D., Otto A., Maaß S., Rohde M., Eckweiler D., Borrero-de Acuña J. M., Jahn M., et al (2018) Iron regulation in *Clostridioides difficile*. Front. Microbiol. 9, 3183 10.3389/fmicb.2018.03183 30619231 PMC6311696

[46] Pohl E., Holmes R. K., and Hol W. G. J. (1999) Crystal structure of the iron-dependent regulator (IdeR) from *Mycobacterium tuberculosis* shows both metal binding sites fully occupied. J. Mol. Biol. 285, 1145–1156 10.1006/jmbi.1998.2339 9887269

[47] Pappenheimer A. M. (1977) Diphtheria toxin. Annu. Rev. Biochem. 46, 69–94 10.1146/annurev.bi.46.070177.000441 20040

[48] Boyd J., Oza M. N., and Murphy J. R. (1990) Molecular cloning and DNA sequence analysis of a diphtheria tox iron-dependent regulatory element (DtxR) from *Corynebacterium diphtheriae*. Proc. Natl. Acad. Sci. U. S. A. 87, 5968–5972 10.1073/pnas.87.15.5968 2116013 PMC54451

[49] Schmitt M. P., and Holmes R. K. (1991) Iron-dependent regulation of diphtheria toxin and siderophore expression by the cloned *Corynebacterium diphtheriae* repressor gene *dtxR* in *C. diphtheriae* C7 strains. Infect. Immun. 59, 1899–1904 10.1128/IAI.59.6.1899-1904.1991 1828057 PMC257940

[50] Tao X., and Murphy J. R. (1994) Determination of the minimal essential nucleotide sequence for diphtheria tox repressor binding by in vitro affinity selection. Proc. Natl. Acad. Sci. U. S. A. 91, 9646–9650 10.1073/pnas.91.20.9646 7937822 PMC44870

[51] White A., Ding X. C., vanderSpek J. C., Murphy J. R., and Ringe D. (1998) Structure of the metal-ion-activated diphtheria toxin repressor tox operator complex. Nature 394, 502–506 10.1038/28893 9697776

[52] Pohl E., Holmes R. K., and Hol W. G. J. (1999) Crystal structure of a cobalt-activated diphtheria toxin repressor-DNA complex reveals a metal-binding SH3-like domain. J. Mol. Biol. 292, 653–667 10.1006/jmbi.1999.3073 10497029

[53] Tao X., Boyd J., and Murphy J. R. (1992) Specific binding of the diphtheria tox regulatory element DtxR to the tox operator requires divalent heavy metal ions and a 9-base pair interrupted palindromic sequence. Proc. Natl. Acad. Sci. U. S. A. 89, 5897–5901 10.1073/pnas.89.13.5897 1631071 PMC49404

[54] Spiering M. M., Ringe D., Murphy J. R., and Marletta M. A. (2003) Metal stoichiometry and functional studies of the diphtheria toxin repressor. Proc. Natl. Acad. Sci. U. S. A. 100, 3808–3813 10.1073/pnas.0737977100 12655054 PMC153003

[55] Ding X., Zeng H., Schiering N., Ringe D., and Murphy J. R. (1996) Identification of the primary metal ion-activation sites of the diphtheria, tox repressor by X-ray crystallography and site-directed mutational analysis. Nat. Struct. Biol. 3, 382–387 10.1038/nsb0496-382 8599765

[56] D'Aquino J. A., Tetenbaum-Novatt J., White A., Berkovitch F., and Ringe D. (2005) Mechanism of metal ion activation of the diphtheria toxin repressor DtxR. Proc. Natl. Acad. Sci. U. S. A. 102, 18408–18413 10.1073/pnas.0500908102 16352732 PMC1317899

[57] Posey J. E., Hardham J. M., Norris S. J., and Gherardini F. C. (1999) Characterization of a manganese-dependent regulatory protein, TroR, from *Treponema pallidum*. Proc. Natl. Acad. Sci. U. S. A. 96, 10887–10892 10.1073/pnas.96.19.10887 10485921 PMC17978

[58] Do H., Makthal N., Chandrangsu P., Olsen R. J., Helmann J. D., Musser J. M., and Kumaraswami M. (2019) Metal sensing and regulation of adaptive responses to manganese limitation by MtsR is critical for group A *streptococcus* virulence. Nucleic Acids Res. 47, 7476–7493 10.1093/nar/gkz524 31188450 PMC6698748

[59] Cheng Y. Q., Yang R. J., Lyu M. Y., Wang S. W., Liu X. C., Wen Y., Song Y., Li J. L., and Chen Z. (2018) IdeR, a DtxR family iron response regulator, controls iron homeostasis, morphological differentiation, secondary metabolism, and the oxidative stress response in *Streptomyces avermitilis*. Appl. Environ. Microbiol. 84, e01503–18 10.1128/aem.01503-18 30194099 PMC6210122

[60] Chao T. C., Becker A., Buhrmester J., Pühler A., and Weidner S. (2004) The *Sinorhizobium meliloti fur* gene regulates, with dependence on Mn(II), transcription of the *sitABCD* operon, encoding a metal-type transporter. J. Bacteriol. 186, 3609–3620 10.1128/JB.186.11.3609-3620.2004 15150249 PMC415740

[61] Rudolph G., Hennecke H., and Fischer H. M. (2006) Beyond the Fur paradigm: iron-controlled gene expression in rhizobia. FEMS Microbiol. Rev. 30, 631–648 10.1111/j.1574-6976.2006.00030.x 16774589

[62] Rodionov D. A., Gelfand M. S., Todd J. D., Curson A. R. J., and Johnston A. W. B. (2006) Computational reconstruction of iron- and manganese-responsive transcriptional networks in α-proteobacteria. PLoS Comp. Biol. 2, 1568–1585 10.1371/journal.pcbi.0020163 17173478 PMC1698941

[63] Hamza I., Chauhan S., Hassett R., and O'Brian M. R. (1998) The bacterial Irr protein is required for coordination of heme biosynthesis with iron availability. J. Biol. Chem. 273, 21669–21674 10.1074/jbc.273.34.21669 9705301

[64] Todd J. D., Wexler M., Sawers G., Yeoman K. H., Poole P. S., and Johnston A. W. B. (2002) RirA, an iron-responsive regulator in the symbiotic bacterium *Rhizobium leguminosarum*. Microbiology 148, 4059–4071 10.1099/00221287-148-12-4059 12480909

[65] Santos J. A., Pereira P. J. B., and Macedo-Ribeiro S. (2015) What a difference a cluster makes: the multifaceted roles of IscR in gene regulation and DNA recognition. Biochim. Biophys. Acta 1854, 1101–1112 10.1016/j.bbapap.2015.01.010 25641558

[66] Pellicer Martinez M. T., Crack J. C., Stewart M. Y. Y., Bradley J. M., Svistunenko D. A., Johnston A. W. B., Cheesman M. R., Todd J. D., and Le Brun N. E. (2019) Mechanisms of iron- and O2-sensing by the [4Fe-4S] cluster of the global iron regulator RirA. Elife 8, e47804 10.7554/eLife.47804 31526471 PMC6748827

[67] Todd J. D., Sawers G., Rodionov D. A., and Johnston A. W. B. (2006) The *Rhizobium leguminosarum* regulator IrrA affects the transcription of a wide range of genes in response to Fe availability. Mol. Genet. Genom. 275, 564–577 10.1007/s00438-006-0115-y 16625355

[68] Yeoman K. H., Curson A. R. J., Todd J. D., Sawers G., and Johnston A. W. B. (2004) Evidence that the *Rhizobium* regulatory protein RirA binds to cis-acting iron-responsive operators (IROs) at promoters of some Fe-regulated genes. Microbiology 150, 4065–4074 10.1099/mic.0.27419-0 15583159

[69] Pellicer Martinez M. T. P., Martinez A. B., Crack J. C., Holmes J. D., Svistunenko D. A., Johnston A. W. B., Cheesman M. R., Todd J. D., and Le Brun N. E. (2017) Sensing iron availability via the fragile [4Fe-4S] cluster of the bacterial transcriptional repressor RirA. Chem. Sci. 8, 8451–8463 10.1039/c7sc02801f 29619193 PMC5863699

[70] Chao T. C., Buhrmester J., Hansmeier N., Pühler A., and Weidner S. (2005) Role of the regulatory gene *rirA* in the transcriptional response of *Sinorhizobium meliloti* to iron limitation. Appl. Environ. Microbiol. 71, 5969–5982 10.1128/AEM.71.10.5969-5982.2005 16204511 PMC1265945

[71] Todd J. D., Sawers G., and Johnston A. W. B. (2005) Proteomic analysis reveals the wide-ranging effects of the novel, iron-responsive regulator RirA in *Rhizobium leguminosarum* bv. *viciae*. Mol. Gen. Genom. 273, 197–206 10.1007/s00438-005-1127-8 15856304

[72] Costa D., Amarelle V., Valverde C., O'Brian M. R., and Fabianoa E. (2017) The Irr and RirA proteins participate in a complex regulatory circuit and act in concert to modulate bacterioferritin expression in *Ensifer meliloti* 1021. Appl. Environ. Microbiol. 83, e00895–17 10.1128/aem.00895-17 28625986 PMC5541210

[73] O'Brian M. R. (2015) Perception and homeostatic control of iron in the *Rhizobia* and related Bacteria. in. Annu. Rev. Microbiol. 69, 229–245 10.1146/annurev-micro-091014-104432 26195304

[74] Qi Z. H., and O'Brian M. R. (2002) Interaction between the bacterial iron response regulator and ferrochelatase mediates genetic control of heme biosynthesis. Mol. Cell 9, 155–162 10.1016/S1097-2765(01)00431-2 11804594

[75] Rudolph G., Semini G., Hauser F., Lindemann A., Friberg M., Hennecke H., and Fischer H. M. (2006) The iron control element, acting in positive and negative control of iron-regulated *Bradyrhizobium japonicum* genes, is a target for the Irr protein. J. Bacteriol. 188, 733–744 10.1128/JB.188.2.733-744.2006 16385063 PMC1347296

[76] Qi Z. H., Hamza I., and O'Brian M. R. (1999) Heme is an effector molecule for iron-dependent degradation of the bacterial iron response regulator (Irr) protein. Proc. Natl. Acad. Sci. U. S. A. 96, 13056–13061 10.1073/pnas.96.23.13056 10557272 PMC23899

[77] Singleton C., White G. F., Todd J. D., Marritt S. J., Cheesman M. R., Johnston A. W. B., and Le Brun N. E. (2010) Heme-responsive DNA binding by the global iron regulator Irr from *Rhizobium leguminosarum*. J. Biol. Chem. 285, 16023–16031 10.1074/jbc.M109.067215 20233710 PMC2871471

[78] Bhubhanil S., Ruangkiattikul N., Niamyim P., Chamsing J., Ngok-Ngam P., Sukchawalit R., and Mongkolsuk S. (2012) Identification of amino acid residues important for the function of *Agrobacterium tumefaciens* Irr protein. FEMS Microbiol. Lett. 335, 68–77 10.1111/j.1574-6968.2012.02638.x 22817265

[79] White G. F., Singleton C., Todd J. D., Cheesman M. R., Johnston A. W. B., and Le Brun N. E. (2011) Heme binding to the second, lower-affinity site of the global iron regulator Irr from *Rhizobium leguminosarum* promotes oligomerization. FEBS J. 278, 2011–2021 10.1111/j.1742-4658.2011.08117.x 21481185

[80] Yang J. H., Ishimori K., and O'Brian M. R. (2005) Two heme binding sites are involved in the regulated degradation of the bacterial iron response regulator (Irr) protein. J. Biol. Chem. 280, 7671–7676 10.1074/jbc.M411664200 15613477

[81] Kobayashi K., Nakagaki M., Ishikawa H., Iwai K., O'Brian M. R., and Ishimori K. (2016) Redox-dependent dynamics in heme-bound bacterial iron response regulator (Irr) protein. Biochemistry 55, 4047–4054 10.1021/acs.biochem.6b00512 27379473

[82] Ratledge C., and Dover L. G. (2000) Iron metabolism in pathogenic bacteria. Annu. Rev. Microbiol. 54, 881–941 10.1146/annurev.micro.54.1.881 11018148

[83] Ellermann M., and Arthur J. C. (2017) Siderophore-mediated iron acquisition and modulation of host-bacterial interactions. Free Radic. Biol. Med. 105, 68–78 10.1016/j.freeradbiomed.2016.10.489 27780750 PMC5401654

[84] Andrews S. C., Robinson A. K., and Rodríguez-Quiñones F. (2003) Bacterial iron homeostasis. FEMS Microbiol. Rev. 27, 215–237 10.1016/S0168-6445(03)00055-X 12829269

[85] Miethke M. (2013) Molecular strategies of microbial iron assimilation: from high-affinity complexes to cofactor assembly systems. Metallomics 5, 15–28 10.1039/c2mt20193c 23192658

[86] Huang W. L., and Wilks A. (2017) Extracellular heme uptake and the challenge of bacterial cell membranes. Annu. Rev. Biochem. 86, 799–823 10.1146/annurev-biochem-060815-014214 28426241

[87] Sheldon J. R., and Heinrichs D. E. (2015) Recent developments in understanding the iron acquisition strategies of Gram positive pathogens. FEMS Microbiol. Rev. 39, 592–630 10.1093/femsre/fuv009 25862688

[88] Lau C. K. Y., Krewulak K. D., and Vogel H. J. (2016) Bacterial ferrous iron transport: the Feo system. FEMS Microbiol. Rev. 40, 273–298 10.1093/femsre/fuv049 26684538

[89] Hider R. C., and Kong X. L. (2010) Chemistry and biology of siderophores. Nat. Prod. Rep. 27, 637–657 10.1039/b906679a 20376388

[90] Schalk I. J., Mislin G. L. A., and Brillet K. (2012) Structure, function and binding selectivity and stereoselectivity of siderophore-iron outer membrane transporters. Curr. Top. Membr. 69, 37–66 10.1016/b978-0-12-394390-3.00002-1 23046646

[91] Furrer J. L., Sanders D. N., Hook-Barnard I. G., and McIntosh M. A. (2002) Export of the siderophore enterobactin in *Escherichia coli*: involvement of a 43 kDa membrane exporter. Mol. Microbiol. 44, 1225–1234 10.1046/j.1365-2958.2002.02885.x 12068807

[92] Imperi F., Tiburzi F., and Visca P. (2009) Molecular basis of pyoverdine siderophore recycling in *Pseudomonas aeruginosa*. Proc. Natl. Acad. Sci. U. S. A. 106, 20440–20445 10.1073/pnas.0908760106 19906986 PMC2787144

[93] Wilson B. R., Bogdan A. R., Miyazawa M., Hashimoto K., and Tsuji Y. (2016) Siderophores in iron metabolism: from mechanism to therapy potential. Trends Mol. Med. 22, 1077–1090 10.1016/j.molmed.2016.10.005 27825668 PMC5135587

[94] McRose D. L., Seyedsayamdost M. R., and Morel F. M. M. (2018) Multiple siderophores: bug or feature? J. Biol. Inorg. Chem. 23, 983–993 10.1007/s00775-018-1617-x 30264174

[95] Schalk I. J., and Guillon L. (2013) Fate of ferrisiderophores after import across bacterial outer membranes: different iron release strategies are observed in the cytoplasm or periplasm depending on the siderophore pathways. Amino Acids 44, 1267–1277 10.1007/s00726-013-1468-2 23443998

[96] Greenwald J., Hoegy F., Nader M., Journet L., Mislin G. L. A., Graumann P. L., and Schalk I. J. (2007) Real time fluorescent resonance energy transfer visualization of ferric pyoverdine uptake in *Pseudomonas aeruginosa*—a role for ferrous iron. J. Biol. Chem. 282, 2987–2995 10.1074/jbc.M609238200 17148441

[97] Smith A. D., and Wilks A. (2012) Extracellular heme uptake and the challenges of bacterial cell membranes. Curr. Top. Membr. 69, 359–392 10.1016/b978-0-12-394390-3.00013-6 23046657 PMC3731948

[98] Braun V., and Hantke K. (2011) Recent insights into iron import by bacteria. Curr. Opin. Chem. Biol. 15, 328–334 10.1016/j.cbpa.2011.01.005 21277822

[99] Létoffé S., Deniau C., Wolff N., Dassa E., Delepelaire P., Lecroisey A., and Wandersman C. (2001) Haemophore-mediated bacterial haem transport: evidence for a common or overlapping site for haem-free and haem-loaded haemophore on its specific outer membrane receptor. Mol. Microbiol. 41, 439–450 10.1046/j.1365-2958.2001.02530.x 11489129

[100] Deniau C., Gilli R., Izadi-Pruneyre N., Létoffé S., Delepierre M., Wandersman C., Briand C., and Lecroisey A. (2003) Thermodynamics of heme binding to the HasA(SM) hemophore: effect of mutations at three key residues for heme uptake. Biochemistry 42, 10627–10633 10.1021/bi030015k 12962486

[101] Krieg S., Huché F., Diederichs K., Izadi-Pruneyre N., Lecroisey A., Wandersman C., Delepelaire P., and Welte W. (2009) Heme uptake across the outer membrane as revealed by crystal structures of the receptor-hemophore complex. Proc. Natl. Acad. Sci. U. S. A. 106, 1045–1050 10.1073/pnas.0809406106 19144921 PMC2633585

[102] Smith A. D., and Wilks A. (2015) Differential contributions of the outer membrane receptors PhuR and HasR to heme acquisition in *Pseudomonas aeruginosa*. J. Biol. Chem. 290, 7756–7766 10.1074/jbc.M114.633495 25616666 PMC4367277

[103] Smith A. D., Modi A. R., Sun S. F., Dawson J. H., and Wilks A. (2015) Spectroscopic determination of distinct heme ligands in outer-membrane receptors PhuR and HasR of *Pseudomonas aeruginosa*. Biochemistry 54, 2601–2612 10.1021/acs.biochem.5b00017 25849630 PMC4627663

[104] Wilks A., and Ikeda-Saito M. (2014) Heme utilization by pathogenic bacteria: not all pathways lead to biliverdin. Acc. Chem. Res. 47, 2291–2298 10.1021/ar500028n 24873177 PMC4139177

[105] Llamas M. A., Imperi F., Visca P., and Lamont I. L. (2014) Cell-surface signaling in *Pseudomonas*: stress responses, iron transport, and pathogenicity. FEMS Microbiol. Rev. 38, 569–597 10.1111/1574-6976.12078 24923658

[106] Sestok A. E., Linkous R. O., and Smith A. T. (2018) Toward a mechanistic understanding of Feo-mediated ferrous iron uptake. Metallomics 10, 887–898 10.1039/c8mt00097b 29953152 PMC6051883

[107] Makui H., Roig E., Cole S. T., Helmann J. D., Gros P., and Cellier M. F. M. (2000) Identification of the *Escherichia coli* K-12 Nramp orthologue (MntH) as a selective divalent metal ion transporter. Mol. Microbiol. 35, 1065–1078 10.1046/j.1365-2958.2000.01774.x 10712688

[108] Grass G., Franke S., Taudte N., Nies D. H., Kucharski L. M., Maguire M. E., and Rensing C. (2005) The metal permease ZupT from *Escherichia coli* is a transporter with a broad substrate spectrum. J. Bacteriol. 187, 1604–1611 10.1128/JB.187.5.1604-1611.2005 15716430 PMC1064025

[109] Perry R. D., Mier I., and Fetherston J. D. (2007) Roles of the Yfe and Feo transporters of *Yersinia pestis* in iron uptake and intracellular growth. Biometals 20, 699–703 10.1007/s10534-006-9051-x 17206386

[110] Katoh H., Hagino N., and Ogawa T. (2001) Iron-binding activity of FutA1 subunit of an ABC-type iron transporter in the cyanobacterium *Synechocystis* sp. strain PCC 6803. Plant Cell Physiol. 42, 823–827 10.1093/pcp/pce106 11522907

[111] Cao J., Woodhall M. R., Alvarez J., Cartron M. L., and Andrews S. C. (2007) EfeUOB (YcdNOB) is a tripartite, acid-induced and CpxAR-regulated, low-pH Fe^2+^ transporter that is cryptic in *Escherichia coli* K-12 but functional in *E. coli* O157: H7. Mol. Microbiol. 65, 857–875 10.1111/j.1365-2958.2007.05802.x 17627767

[112] Kammler M., Schön C., and Hantke K. (1993) Characterization of the ferrous iron uptake system of *Escherichia coli*. J. Bacteriol. 175, 6212–6219 10.1128/jb.175.19.6212-6219.1993 8407793 PMC206716

[113] Marlovits T. C., Haase W., Herrmann C., Aller S. G., and Unger V. M. (2002) The membrane protein FeoB contains an intramolecular G protein essential for Fe(II) uptake in bacteria. Proc. Natl. Acad. Sci. U. S. A. 99, 16243–16248 10.1073/pnas.242338299 12446835 PMC138596

[114] Seyedmohammad S., Born D., and Venter H. (2014) Expression, purification and functional reconstitution of FeoB, the ferrous iron transporter from *Pseudomonas aeruginosa*. Protein Expr. Purif. 101, 138–145 10.1016/j.pep.2014.06.012 24993789

[115] Smith A. T., and Sestok A. E. (2018) Expression and purification of functionally active ferrous iron transporter FeoB from *Klebsiella pneumoniae*. Protein Expr. Purif. 142, 1–7 10.1016/j.pep.2017.09.007 28941825

[116] Ash M. R., Guilfoyle A., Clarke R. J., Guss M., Maher M. J., and Jormakka M. (2010) Potassium-activated GTPase reaction in the G protein-coupled ferrous iron transporter B. J. Biol. Chem. 285, 14594–14602 10.1074/jbc.M110.111914 20220129 PMC2863241

[117] Eng E. T., Jalilian A. R., Spasov K. A., and Unger V. M. (2008) Characterization of a novel prokaryotic GDP dissociation inhibitor domain from the G protein coupled membrane protein FeoB. J. Mol. Biol. 375, 1086–1097 10.1016/j.jmb.2007.11.027 18068722 PMC2266681

[118] Cartron M. L., Maddocks S., Gillingham P., Craven C. J., and Andrews S. C. (2006) Feo—transport of ferrous iron into bacteria. Biometals 19, 143–157 10.1007/s10534-006-0003-2 16718600

[119] Su Y. C., Chin K. H., Hung H. C., Shen G. H., Wang A. H. J., and Chou S. H. (2010) Structure of *Stenotrophomonas maltophilia* FeoA complexed with zinc: a unique prokaryotic SH3-domain protein that possibly acts as a bacterial ferrous iron-transport activating factor. Acta Crystallogr. F 66, 636–642 10.1107/S1744309110013941 20516589 PMC2882759

[120] Hung K. W., Juan T. H., Hsu Y. L., and Huang T. H. (2012) NMR structure note: the ferrous iron transport protein C (FeoC) from *Klebsiella pneumoniae*. J. Biomol. NMR 53, 161–165 10.1007/s10858-012-9633-6 22580893

[121] Hung K. W., Tsai J. Y., Juan T. H., Hsu Y. L., Hsiao C. D., and Huang T. H. (2012) Crystal structure of the *Klebsiella pneumoniae* NFeoB/FeoC complex and roles of FeoC in regulation of Fe^2+^ transport by the bacterial Feo system. J. Bacteriol. 194, 6518–6526 10.1128/JB.01228-12 23024345 PMC3497523

[122] Maddocks S. E., and Oyston P. C. F. (2008) Structure and function of the LysR-type transcriptional regulator (LTTR) family proteins. Microbiology 154, 3609–3623 10.1099/mic.0.2008/022772-0 19047729

[123] Skaar E. P., Gaspar A. H., and Schneewind O. (2004) IsdG and IsdI, heme-degrading enzymes in the cytoplasm of *Staphylococcus aureus*. J. Biol. Chem. 279, 436–443 10.1074/jbc.M307952200 14570922

[124] Skaar E. P., Gaspar A. H., and Schneewind O. (2006) *Bacillus anthracis* IsdG, a heme-degrading monooxygenase. J. Bacteriol. 188, 1071–1080 10.1128/JB.188.3.1071-1080.2006 16428411 PMC1347327

[125] Mazmanian S. K., Skaar E. P., Gaspar A. H., Humayun M., Gornicki P., Jelenska J., Joachmiak A., Missiakas D. M., and Schneewind O. (2003) Passage of heme-iron across the envelope of *Staphylococcus aureus*. Science 299, 906–909 10.1126/science.1081147 12574635

[126] Marraffini L. A., Ton-That H., Zong Y. N., Narayana S. V. L., and Schneewind O. (2004) Anchoring of surface proteins to the cell wall of *Staphylococcus aureus*: a conserved arginine residue is required for efficient catalysis of sortase A. J. Biol. Chem. 279, 37763–37770 10.1074/jbc.M405282200 15247224

[127] Pilpa R. M., Robson S. A., Villareal V. A., Wong M. L., Phillips M., and Clubb R. T. (2009) Functionally distinct NEAT (NEAr Transporter) domains within the *Staphylococcus aureus* IsdH/HarA protein extract heme from methemoglobin. J. Biol. Chem. 284, 1166–1176 10.1074/jbc.M806007200 18984582 PMC2613621

[128] Muryoi N., Tiedemann M. T., Pluym M., Cheung J., Heinrichs D. E., and Stillman M. J. (2008) Demonstration of the iron-regulated surface determinant (Isd) heme transfer pathway in *Staphylococcus aureus*. J. Biol. Chem. 283, 28125–28136 10.1074/jbc.M802171200 18676371 PMC2661384

[129] Moriwaki Y., Terada T., Caaveiro J. M. M., Takaoka Y., Hamachi I., Tsumoto K., and Shimizu K. (2013) Heme binding mechanism of structurally similar iron-regulated surface determinant near transporter domains of *Staphylococcus aureus* exhibiting different affinities for heme. Biochemistry 52, 8866–8877 10.1021/bi4008325 24245481

[130] Williams R. J. P. (1982) Free manganese(II) and iron(II) cations can act as intracellular cell controls. FEBS Lett. 140, 3–10 10.1016/0014-5793(82)80508-5 7084455

[131] Wofford J. D., Bolaji N., Dziuba N., Outten F. W., and Lindahl P. A. (2019) Evidence that a respiratory shield in *Escherichia coli* protects a low-molecular-mass Fe^2+^ pool from O_2_-dependent oxidation. J. Biol. Chem. 294, 50–62 10.1074/jbc.RA118.005233 30337367 PMC6322884

[132] Beaufay F., Quarles E., Franz A., Katamanin O., Wholey W.-Y., and Jakob U. (2020) Polyphosphate functions *in vivo* as an iron chelator and Fenton reaction inhibitor. mBio 11, e01017 10.1128/mBio.01017-20 32723918 PMC7387796

[133] Hristova D., Wu C. H., Jiang W., Krebs C., and Stubbe J. (2008) Importance of the maintenance pathway in the regulation of the activity of *Escherichia coli* ribonucleotide reductase. Biochemistry 47, 3989–3999 10.1021/bi702408k 18314964 PMC2801593

[134] Bradley J. M., Moore G. R., and Le Brun N. E. (2017) Diversity of Fe^2+^ entry and oxidation in ferritins. Curr. Opin. Chem. Biol. 37, 122–128 10.1016/j.cbpa.2017.02.027 28314217

[135] Theil E. C., Behera R. K., and Tosha T. (2013) Ferritins for chemistry and for life. Coord. Chem. Rev. 257, 579–586 10.1016/j.ccr.2012.05.013 23470857 PMC3587046

[136] Andrews S. C. (2010) The Ferritin-like superfamily: evolution of the biological iron storeman from a rubrerythrin-like ancestor. Biochim. Biophys. Acta 1800, 691–705 10.1016/j.bbagen.2010.05.010 20553812

[137] Zeth K., Offermann S., Essen L. O., and Oesterhelt D. (2004) Iron-oxo clusters biomineralizing on protein surfaces: structural analysis of *Halobacterium salinarum* DpsA in its low- and high-iron states. Proc. Natl. Acad. Sci. U. S. A. 101, 13780–13785 10.1073/pnas.0401821101 15365182 PMC518833

[138] Stillman T. J., Hempstead P. D., Artymiuk P. J., Andrews S. C., Hudson A. J., Treffry A., Guest J. R., and Harrison P. M. (2001) The high-resolution X-ray crystallographic structure of the ferritin (EcFtnA) of *Escherichia coli*: comparison with human H ferritin (HuHF) and the structures of the Fe^3+^ and Zn^2+^ derivatives. J. Mol. Biol. 307, 587–603 10.1006/jmbi.2001.4475 11254384

[139] Ohtomo H., Ohtomo M., Sato D., Kurobe A., Sunato A., Matsumura Y., Kihara H., Fujiwara K., and Ikeguchi M. (2015) A physicochemical and mutational analysis of intersubunit interactions of *Escherichia coli* ferritin A. Biochemistry 54, 6243–6251 10.1021/acs.biochem.5b00723 26399896

[140] Theil E. C. (2011) Ferritin protein nanocages use ion channels, catalytic sites, and nucleation channels to manage iron/oxygen chemistry. Curr. Opin. Chem. Biol. 15, 304–311 10.1016/j.cbpa.2011.01.004 21296609 PMC3074017

[141] Bradley J. M., Pullin J., Moore G. R., Svistunenko D. A., Hemmings A. M., and Le Brun N. E. (2020) Routes of iron entry into, and exit from, the catalytic ferroxidase sites of the prokaryotic ferritin SynFtn. Dalton Trans. 49, 1545–1554 10.1039/c9dt03570b 31930254

[142] Wong S. G., Grigg J. C., Le Brun N. E., Moore G. R., Murphy M. E. P., and Mauk A. G. (2015) The B-type channel is a major route for iron entry into the ferroxidase center and central cavity of bacterioferritin. J. Biol. Chem. 290, 3732–3739 10.1074/jbc.M114.623082 25512375 PMC4319037

[143] Ebrahimi K. H., Hagedoorn P. L., and Hagen W. R. (2015) Unity in the biochemistry of the iron-storage proteins ferritin and bacterioferritin. Chem. Rev. 115, 295–326 10.1021/cr5004908 25418839

[144] Williams S. M., and Chatterji D. (2017) Flexible aspartates propel iron to the ferroxidation sites along pathways stabilized by a conserved arginine in Dps proteins from *Mycobacterium smegmatis*. Metallomics 9, 685–698 10.1039/c7mt00008a 28418062

[145] Behera R. K., and Theil E. C. (2014) Moving Fe^2+^ from ferritin ion channels to catalytic OH centers depends on conserved protein cage carboxylates. Proc. Natl. Acad. Sci. U. S. A. 111, 7925–7930 10.1073/pnas.1318417111 24843174 PMC4050572

[146] Masuda T., Goto F., Yoshihara T., and Mikami B. (2010) The universal mechanism for iron translocation to the ferroxidase site in ferritin, which is mediated by the well conserved transit site. Biochem. Biophys. Res. Commun. 400, 94–99 10.1016/j.bbrc.2010.08.017 20705053

[147] Bradley J. M., Moore G. R., and Le Brun N. E. (2014) Mechanisms of iron mineralization in ferritins: one size does not fit all. J. Biol. Inorg. Chem. 19, 775–785 10.1007/s00775-014-1136-3 24748222

[148] Haikarainen T., and Papageorgiou A. C. (2010) Dps-like proteins: structural and functional insights into a versatile protein family. Cell. Mol. Life Sci. 67, 341–351 10.1007/s00018-009-0168-2 19826764 PMC11115558

[149] Pourciau C., Pannuri A., Potts A., Yakhnin H., Babitzke P., and Romeo T. (2019) Regulation of iron storage by CsrA supports exponential growth of *Escherichia coli*. Mbio 10, e01034–19 10.1128/mBio.01034-19 31387901 PMC6686035

[150] Laulhère J. P., Labouré A. M., Van Wuytswinkel O., Gagnon J., and Briat J. F. (1992) Purification, characterization and function of bacterioferritin from the cyanobacterium *Synechocystis* PCC-6803. Biochemistry 281, 785–793 10.1042/bj2810785 1536655 PMC1130759

[151] Shcolnick S., Summerfield T. C., Reytman L., Sherman L. A., and Keren N. (2009) The mechanism of iron homeostasis in the unicellular cyanobacterium *Synechocystis* sp PCC 6803 and its relationship to oxidative stress. Plant Physiol. 150, 2045–2056 10.1104/pp.109.141853 19561120 PMC2719147

[152] Treffry A., Zhao Z. W., Quail M. A., Guest J. R., and Harrison P. M. (1998) How the presence of three iron binding sites affects the iron storage function of the ferritin (EcFtnA) of *Escherichia coli*. FEBS Lett. 432, 213–218 10.1016/S0014-5793(98)00867-9 9720927

[153] Bou-Abdallah F., Yang H., Awomolo A., Cooper B., Woodhall M. R., Andrews S. C., and Chasteen N. D. (2014) Functionality of the three-site ferroxidase center of *Escherichia coli* bacterial ferritin (EcFtnA). Biochemistry 53, 483–495 10.1021/bi401517f 24380371 PMC3951517

[154] Ebrahimi K. H., Hagedoorn P. L., and Hagen W. R. (2013) A conserved tyrosine in ferritin is a molecular capacitor. Chembiochem 14, 1123–1133 10.1002/cbic.201300149 23737293

[155] Mohanty A., Subhadarshanee B., Barman P., Mahapatra C., Aishwarya B., and Behera R. K. (2019) Iron mineralizing bacterioferritin A from *Mycobacterium tuberculosis* exhibits unique catalase-Dps-like dual activities. Inorg. Chem. 58, 4741–4752 10.1021/acs.inorgchem.8b02758 30920210

[156] Levi S., Luzzago A., Cesareni G., Cozzi A., Franceschinelli F., Albertini A., and Arosio P. (1988) Mechanism of ferritin iron uptake: activity of the H-chain and deletion mapping of the ferro-oxidase site. A study of iron uptake and ferro-oxidase activity of human-liver, recombinant H-chain ferritins, and of 2 H-chain deletion mutants. J. Biol. Chem. 263, 18086–18092 3192527

[157] Ingrassia R., Gerardi G., Biasiotto G., and Arosio P. (2006) Mutations of ferritin H chain C-terminus produced by nucleotide insertions have altered stability and functional properties. J. Biochem. 139, 881–885 10.1093/jb/mvj101 16751596

[158] Andrews S. C., Le Brun N. E., Barynin V., Thomson A. J., Moore G. R., Guest J. R., and Harrison P. M. (1995) Site-directed replacement of the coaxial heme ligands of bacterioferritin generates heme-free variants. J. Biol. Chem. 270, 23268–23274 10.1074/jbc.270.40.23268 7559480

[159] Yasmin S., Andrews S. C., Moore G. R., and Le Brun N. E. (2011) A new role for heme, facilitating release of iron from the bacterioferritin iron biomineral. J. Biol. Chem. 286, 3473–3483 10.1074/jbc.M110.175034 21106523 PMC3030353

[160] Yao H. L., Wang Y., Lovell S., Kumar R., Ruvinsky A. M., Battaile K. P., Vakser I. A., and Rivera M. (2012) The structure of the BfrB-Bfd complex reveals protein-protein interactions enabling iron release from bacterioferritin. J. Am. Chem. Soc. 134, 13470–13481 10.1021/ja305180n 22812654 PMC3428730

[161] Crow A., Lawson T. L., Lewin A., Moore G. R., and Le Brun N. E. (2009) Structural basis for iron mineralization by bacterioferritin. J. Am. Chem. Soc. 131, 6808–6813 10.1021/ja8093444 19391621

[162] Kwak Y., Schwartz J. K., Huang V. W., Boice E., Kurtz D. M., and Solomon E. I. (2015) CD/MCD/VTVH-MCD studies of *Escherichia coli* bacterioferritin support a binuclear iron cofactor site. Biochemistry 54, 7010–7018 10.1021/acs.biochem.5b01033 26551523

[163] Baaghil S., Lewin A., Moore G. R., and Le Brun N. E. (2003) Core formation in *Escherichia coli* bacterioferritin requires a functional. Biochemistry 42, 14047–14056 10.1021/bi035253u 14636073

[164] Bradley J. M., Svistunenko D. A., Lawson T. L., Hemmings A. M., Moore G. R., and Le Brun N. E. (2015) Three aromatic residues are required for electron transfer during iron mineralization in bacterioferritin. Angew. Chem. Int. Ed. 54, 14763–14767 10.1002/anie.201507486 26474305 PMC4691338

[165] Bradley J. M., Svistunenko D. A., Moore G. R., and Le Brun N. E. (2017) Tyr25, Tyr58 and Trp133 of *Escherichia coli* bacterioferritin transfer electrons between iron in the central cavity and the ferroxidase centre. Metallomics 9, 1421–1428 10.1039/c7mt00187h 28914315

[166] Bou-Abdallah F., Lewin A. C., Le Brun N. E., Moore G. R., and Chasteen N. D. (2002) Iron detoxification properties of *Escherichia coli* bacterioferritin: attenuation of oxyradical chemistry. J. Biol. Chem. 277, 37064–37069 10.1074/jbc.M205712200 12124394

[167] Weeratunga S. K., Lovell S., Yao H. L., Battaile K. P., Fischer C. J., Gee C. E., and Rivera M. (2010) Structural studies of bacterioferritin B from *Pseudomonas aeruginosa* suggest a gating mechanism for iron uptake via the ferroxidase center. Biochemistry 49, 1160–1175 10.1021/bi9015204 20067302 PMC2852880

[168] Abdul-Tehrani H., Hudson A. J., Chang Y. S., Timms A. R., Hawkins C., Williams J. M., Harrison P. M., Guest J. R., and Andrews S. C. (1999) Ferritin mutants of *Escherichia coli* are iron deficient and growth impaired, and *fur* mutants are iron deficient. J. Bacteriol. 181, 1415–1428 10.1128/JB.181.5.1415-1428.1999 10049371 PMC93529

[169] Eshelman K., Yao H. L., Hewage A., Deay J. J., Chandler J. R., and Rivera M. (2017) Inhibiting the BfrB: Bfd interaction in *Pseudomonas aeruginosa* causes irreversible iron accumulation in bacterioferritin and iron deficiency in the bacterial cytosol. Metallomics 9, 646–659 10.1039/c7mt00042a 28318006 PMC5494978

[170] Velayudhan J., Castor M., Richardson A., Main-Hester K. L., and Fang F. C. (2007) The role of ferritins in the physiology of *Salmonella enterica* sv. *Typhimurium*: a unique role for ferritin B in iron-sulphur cluster repair and virulence. Mol. Microbiol. 63, 1495–1507 10.1111/j.1365-2958.2007.05600.x 17302823

[171] He X., Jiang H. W., Chen H., Zhang H. N., Liu Y., Xu Z. W., Wu F. L., Guo S. J., Hou J. L., Yang M. K., Yan W., Deng J. Y., Bi L. J., Zhang X. E., and Tao S. C. (2017) Systematic identification of *Mycobacterium tuberculosis* effectors reveals that BfrB suppresses innate immunity. Mol. Cell. Proteomics 16, 2243–2253 10.1074/mcp.RA117.000296 29018126 PMC5724184

[172] Khare G., Nangpal P., and Tyagi A. K. (2017) Differential roles of iron storage proteins in maintaining the iron homeostasis in *Mycobacterium tuberculosis*. PLoS ONE 12, e0169545 10.1371/journal.pone.0169545 28060867 PMC5218490

[173] Figueiredo M. C. O., Lobo S. A. L., Carita J. N., Nobre L. S., and Saraiva L. M. (2012) Bacterioferritin protects the anaerobe *Desulfovibrio vulgaris* Hildenborough against oxygen. Anaerobe 18, 454–458 10.1016/j.anaerobe.2012.06.001 22706208

[174] Altuvia S., Almirón M., Huisman G., Kolter R., and Storz G. (1994) The Dps promoter is activated by OxyR during growth and by IHF and a σS in stationary phase. Mol. Microbiol. 13, 265–272 10.1111/j.1365-2958.1994.tb00421.x 7984106

[175] Almiron M., Link A. J., Furlong D., and Kolter R. (1992) A novel DNA-binding protein with regulatory and protective roles in starved *Escherichia coli*. Genes Dev. 6, 2646–2654 10.1101/gad.6.12b.2646 1340475

[176] Azam T. A., and Ishihama A. (1999) Twelve species of the nucleoid-associated protein from *Escherichia coli*—sequence recognition specificity and DNA binding affinity. J. Biol. Chem. 274, 33105–33113 10.1074/jbc.274.46.33105 10551881

[177] Frenkiel-Krispin D., Ben-Avraham I., Englander J., Shimoni E., Wolf S. G., and Minsky A. (2004) Nucleoid restructuring in stationary-state bacteria. Mol. Microbiol. 51, 395–405 10.1046/j.1365-2958.2003.03855.x 14756781

[178] Wolf S. G., Frenkiel D., Arad T., Finkel S. E., Kolter R., and Minsky A. (1999) DNA protection by stress-induced biocrystallization. Nature 400, 83–85 10.1038/21918 10403254

[179] Grant R. A., Filman D. J., Finkel S. E., Kolter R., and Hogle J. M. (1998) The crystal structure of Dps, a ferritin homolog that binds and protects DNA. Nat. Struct. Biol. 5, 294–303 10.1038/nsb0498-294 9546221

[180] Ceci P., Cellai S., Falvo E., Rivetti C., Rossi G. L., and Chiancone E. (2004) DNA condensation and self-aggregation of *Escherichia coli* Dps are coupled phenomena related to the properties of the N-terminus. Nucleic Acids Res. 32, 5935–5944 10.1093/nar/gkh915 15534364 PMC528800

[181] Karas V. O., Westerlaken I., and Meyer A. S. (2015) The DNA-binding protein from starved cells (Dps) utilizes dual functions to defend cells against multiple stresses. J. Bacteriol. 197, 3206–3215 10.1128/JB.00475-15 26216848 PMC4560292

[182] Tsou C. C., Chiang-Ni C., Lin Y. S., Chuang W. J., Lin M. T., Liu C. C., and Wu J. J. (2010) Oxidative stress and metal ions regulate a ferritin-like gene, dpr, in *Streptococcus pyogenes*. Int. J. Med. Microbiol. 300, 259–264 10.1016/j.ijmm.2009.09.002 19879189

[183] Ulijasz A. T., Andes D. R., Glasner J. D., and Weisblum B. (2004) Regulation of iron transport in *Streptococcus pneumoniae* by RitR, an orphan response regulator. J. Bacteriol. 186, 8123–8136 10.1128/JB.186.23.8123-8136.2004 15547286 PMC529065

[184] Ilari A., Stefanini S., Chiancone E., and Tsernoglou D. (2000) The dodecameric ferritin from *Listeria innocua* contains a novel intersubunit iron-binding site. Nat. Struct. Biol. 7, 38–43 10.1038/71236 10625425

[185] Kauko A., Pulliainen A. T., Haataja S., Meyer-Klaucke W., Finne J., and Papageorgiou A. C. (2006) Iron incorporation in *Streptococcus suis* Dps-like peroxide resistance protein Dpr requires mobility in the ferroxidase center and leads to the formation of a ferrihydrite-like core. J. Mol. Biol. 364, 97–109 10.1016/j.jmb.2006.08.061 16997323

[186] Roy S., Gupta S., Das S., Sekar K., Chatterji D., and Vijayan M. (2004) X-ray analysis of *Mycobacterium smegmatis* Dps and a comparative study involving other Dps and Dps-like molecules. J. Mol. Biol. 339, 1103–1113 10.1016/j.jmb.2004.04.042 15178251

[187] Ren B., Tibbelin G., Kajino T., Asami O., and Ladenstein R. (2003) The multi-layered structure of Dps with a novel di-nuclear ferroxidase center. J. Mol. Biol. 329, 467–477 10.1016/S0022-2836(03)00466-2 12767829

[188] Su M. H., Cavallo S., Stefanini S., Chiancone E., and Chasteen N. D. (2005) The so-called *Listeria innocua* ferritin is a Dps protein. Iron incorporation, detoxification, and DNA protection properties. Biochemistry 44, 5572–5578 10.1021/bi0472705 15823015

[189] Zhao G. H., Ceci P., Ilari A., Giangiacomo L., Laue T. M., Chiancone E., and Chasteen N. D. (2002) Iron and hydrogen peroxide detoxification properties of DNA-binding protein from starved cells—a ferritin-like DNA-binding protein of *Escherichia coli*. J. Biol. Chem. 277, 27689–27696 10.1074/jbc.M202094200 12016214

[190] Bradley J. M., Svistunenko D. A., Pullin J., Hill N., Stuart R. K., Palenik B., Wilson M. T., Hemmings A. M., Moore G. R., and Le Brun N. E. (2019) Reaction of O_2_ with a diiron protein generates a mixed-valent Fe^2+^/Fe^3+^ center and peroxide. Proc. Natl. Acad. Sci. U. S. A. 116, 2058–2067 10.1073/pnas.1809913116 30659147 PMC6369749

[191] Ekman M., Sandh G., Nenninger A., Oliveira P., and Stensjö K. (2014) Cellular and functional specificity among ferritin-like proteins in the multicellular cyanobacterium *Nostoc punctiforme*. Environ. Microbiol. 16, 829–844 10.1111/1462-2920.12233 23992552

[192] Li X., Sandh G., Nenninger A., Muro-Pastor A. M., and Stensjo K. (2015) Differential transcriptional regulation of orthologous *dps* genes from two closely related heterocyst-forming cyanobacteria. FEMS Microbiol Lett. 362, fnv017 10.1093/femsle/fnv017 25663155

[193] Meeks J. C., Elhai J., Thiel T., Potts M., Larimer F., Lamerdin J., Predki P., and Atlas R. (2001) An overview of the genome of *Nostoc punctiforme*, a multicellular, symbiotic cyanobacterium. Photosynth. Res. 70, 85–106 10.1023/A:1013840025518 16228364

[194] Moparthi V. K., Li X., Vavitsas K., Dzhygyr I., Sandh G., Magnuson A., and Stensjö K. (2016) The two Dps proteins, NpDps2 and NpDps5, are involved in light-induced oxidative stress tolerance in the N_2_-fixing cyanobacterium *Nostoc punctiforme*. Biochim. Biophys. Acta 1857, 1766–1776 10.1016/j.bbabio.2016.08.003 27528559

[195] Howe C., Ho F., Nenninger A., Raleiras P., and Stensjö K. (2018) Differential biochemical properties of three canonical Dps proteins from the cyanobacterium *Nostoc punctiforme* suggest distinct cellular functions. J. Biol. Chem. 293, 16635–16646 10.1074/jbc.RA118.002425 30171072 PMC6204913

[196] Sato N., Moriyama T., Toyoshima M., Mizusawa M., and Tajima N. (2012) The all0458/lti46.2 gene encodes a low temperature-induced Dps protein homologue in the cyanobacteria *Anabaena* sp. PCC 7120 and *Anabaena variabilis* M3. Microbiology 158, 2527–2536 10.1099/mic.0.060657-0 22837304

[197] Li X., Mustila H., Magnuson A., and Stensjö K. (2018) Homologous overexpression of NpDps2 and NpDps5 increases the tolerance for oxidative stress in the multicellular cyanobacterium *Nostoc punctiforme*. FEMS Microbiol. Lett. 365, fny198 10.1093/femsle/fny198 30107525 PMC6116882

[198] Howe C., Moparthi V. K., Ho F. M., Persson K., and Stensjö K. (2019) The Dps4 from *Nostoc punctiforme* ATCC 29133 is a member of His-type FOC containing Dps protein class that can be broadly found among cyanobacteria. PLoS ONE 14, e0218300 10.1371/journal.pone.0218300 31369577 PMC6675082

[199] Moparthi V. K., Moparthi S. B., Howe C., Raleiras P., Wenger J., and Stensjo K. (2019) Structural diffusion properties of two atypical Dps from the cyanobacterium *Nostoc punctiforme* disclose interactions with ferredoxins and DNA. Biochim. Biophys. Acta 1860, 148063 10.1016/j.bbabio.2019.148063 31419396

[200] Giessen T. W. (2016) Encapsulins: microbial nanocompartments with applications in biomedicine, nanobiotechnology and materials science. Curr. Opin. Chem. Biol. 34, 1–10 10.1016/j.cbpa.2016.05.013 27232770

[201] Sutter M., Boehringer D., Gutmann S., Günther S., Prangishvili D., Loessner M. J., Stetter K. O., Weber-Ban E., and Ban N. (2008) Structural basis of enzyme encapsulation into a bacterial nanocompartment. Nat. Struct. Mol. Biol. 15, 939–947 10.1038/nsmb.1473 19172747

[202] Akita F., Chong K. T., Tanaka H., Yamashita E., Miyazaki N., Nakaishi Y., Suzuki M., Namba K., Ono Y., Tsukihara T., and Nakagawa A. (2007) The crystal structure of a virus-like particle from the hyperthermophilic archaeon *Pyrococcus furiosus* provides insight into the evolution of viruses. J. Mol. Biol. 368, 1469–1483 10.1016/j.jmb.2007.02.075 17397865

[203] Giessen T. W., Orlando B. J., Verdegaal A. A., Chambers M. G., Gardener J., Bell D. C., Birrane G., Liao M. F., and Silver P. A. (2019) Large protein organelles form a new iron sequestration system with high storage capacity. Elife 8, e46070 10.7554/eLife.46070 31282860 PMC6668986

[204] Contreras H., Joens M. S., McMath L. M., Le V. P., Tullius M. V., Kimmey J. M., Bionghi N., Horwitz M. A., Fitzpatrick J. A. J., and Goulding C. W. (2014) Characterization of a *Mycobacterium tuberculosis* nanocompartment and its potential cargo proteins. J. Biol. Chem. 289, 18279–18289 10.1074/jbc.M114.570119 24855650 PMC4140288

[205] He D. D., Hughes S., Vanden-Hehir S., Georgiev A., Altenbach K., Tarrant E., Mackay C. L., Waldron K. J., Clarke D. J., and Marles-Wright J. (2016) Structural characterization of encapsulated ferritin provides insight into iron storage in bacterial nanocompartments. Elife 5, e18972 10.7554/eLife.18972 27529188 PMC5012862

[206] He D. D., Piergentili C., Ross J., Tarrant E., Tuck L. R., Mackay C. L., McIver Z., Waldron K. J., Clarke D. J., and Marles-Wright J. (2019) Conservation of the structural and functional architecture of encapsulated ferritins in bacteria and archaea. Biochem. J. 476, 975–989 10.1042/BCJ20180922 30837306

[207] McHugh C. A., Fontana J., Nemecek D., Cheng N. Q., Aksyuk A. A., Heymann J. B., Winkler D. C., Lam A. S., Wall J. S., Steven A. C., and Hoiczyk E. (2014) A virus capsid-like nanocompartment that stores iron and protects bacteria from oxidative stress. EMBO J. 33, 1896–1911 10.15252/embj.201488566 25024436 PMC4195785

[208] Giessen T. W., and Silver P. A. (2017) Widespread distribution of encapsulin nanocompartments reveals functional diversity. Nat. Microbiol. 2, 17029 10.1038/nmicrobiol.2017.29 28263314

[209] Pi H. L., and Helmann J. D. (2017) Ferrous iron efflux systems in bacteria. Metallomics 9, 840–851 10.1039/c7mt00112f 28604884 PMC5675029

[210] Guan G. H., Pinochet-Barros A., Gaballa A., Patel S. J., Argüello J. M., and Helmann J. D. (2015) PfeT, a P_1B4_-type ATPase, effluxes ferrous iron and protects *Bacillus subtilis* against iron intoxication. Mol. Microbiol. 98, 787–803 10.1111/mmi.13158 26261021 PMC4648274

[211] Pi H. L., Patel S. J., Argüello J. M., and Helmann J. D. (2016) The *Listeria monocytogenes* Fur-regulated virulence protein FrvA is an Fe(II) efflux P_1B4_-type ATPase. Mol. Microbiol. 100, 1066–1079 10.1111/mmi.13368 26946370 PMC4914386

[212] Patel S. J., Lewis B. E., Long J. E., Nambi S., Sassetti C. M., Stemmler T. L., and Argüello J. M. (2016) Fine-tuning of substrate affinity leads to alternative roles of *Mycobacterium tuberculosis* Fe^2+^-ATPases. J. Biol. Chem. 291, 11529–11539 10.1074/jbc.M116.718239 27022029 PMC4882424

[213] VanderWal A. R., Makthal N., Pinochet-Barros A., Helmann J. D., Olsen R. J., and Kumaraswami M. (2017) Iron efflux by PmtA is critical for oxidative stress resistance and contributes significantly to group A *Streptococcus* virulence. Infect. Immun. 85, e00091–17 10.1128/iai.00091-17 28348051 PMC5442632

[214] Turner A. G., Djoko K. Y., Ong C. L. Y., Barnett T. C., Walker M. J., and McEwan A. G. (2019) Group A *Streptococcus* co-ordinates manganese import and iron efflux in response to hydrogen peroxide stress. Biochem. J. 476, 595–611 10.1042/BCJ20180902 30670571

[215] Zielazinski E. L., González-Guerrero M., Subramanian P., Stemmler T. L., Argüello J. M., and Rosenzweig A. C. (2013) *Sinorhizobium meliloti* Nia is a P_1B5_-ATPase expressed in the nodule during plant symbiosis and is involved in Ni and Fe transport. Metallomics 5, 1614–1623 10.1039/c3mt00195d 24056637 PMC3838458

[216] Grass G., Otto M., Fricke B., Haney C. J., Rensing C., Nies D. H., and Munkelt D. (2005) FieF (YiiP) from *Escherichia coli* mediates decreased cellular accumulation of iron and relieves iron stress. Arch. Microbiol. 183, 9–18 10.1007/s00203-004-0739-4 15549269

[217] Salusso A., and Raimunda D. (2017) Defining the roles of the cation diffusion facilitators in Fe^2+^/Zn^2+^ homeostasis and establishment of their participation in virulence in *Pseudomonas aeruginosa*. Front. Cell. Infect. Microbiol. 7, 84 10.3389/fcimb.2017.00084 28373967 PMC5357649

[218] Bennett B. D., Brutinel E. D., and Gralnick J. A. (2015) A ferrous iron exporter mediates iron resistance in *Shewanella oneidensis* MR-1. Appl. Environ. Microbiol. 81, 7938–7944 10.1128/AEM.02835-15 26341213 PMC4616933

[219] Frawley E. R., Crouch M. L. V., Bingham-Ramos L. K., Robbins H. F., Wang W. L., Wright G. D., and Fang F. C. (2013) Iron and citrate export by a major facilitator superfamily pump regulates metabolism and stress resistance in *Salmonella Typhimurium*. Proc. Natl. Acad. Sci. U. S. A. 110, 12054–12059 10.1073/pnas.1218274110 23821749 PMC3718157

[220] Sankari S., and O'Brian M. R. (2014) A bacterial iron exporter for maintenance of iron homeostasis. J. Biol. Chem. 289, 16498–16507 10.1074/jbc.M114.571562 24782310 PMC4047416

[221] Ruangkiattikul N., Bhubhanil S., Chamsing J., Niamyim P., Sukchawalit R., and Mongkolsuk S. (2012) *Agrobacterium tumefaciens* membrane-bound ferritin plays a role in protection against hydrogen peroxide toxicity and is negatively regulated by the iron response regulator. FEMS Microbiol. Lett. 329, 87–92 10.1111/j.1574-6968.2012.02509.x 22268462

[222] Hagen W. R., Hagedoorn P. L., and Ebrahimi K. H. (2017) The workings of ferritin: a crossroad of opinions. Metallomics 9, 595–605 10.1039/c7mt00124j 28573266

[223] Clarke T. E., Ku S. Y., Dougan D. R., Vogel H. J., and Tari L. W. (2000) The structure of the ferric siderophore binding protein FhuD complexed with gallichrome. Nat. Struct. Biol. 7, 287–291 10.1038/74048 10742172

[224] Mattle D., Zeltina A., Woo J. S., Goetz B. A., and Locher K. P. (2010) Two stacked heme molecules in the binding pocket of the periplasmic heme-binding protein HmuT from *Yersinia pestis*. J. Mol. Biol. 404, 220–231 10.1016/j.jmb.2010.09.005 20888343

[225] Woo J. S., Zeltina A., Goetz B. A., and Locher K. P. (2012) X-ray structure of the *Yersinia pestis* heme transporter HmuUV. Nat. Struct. Mol. Biol. 19, 1310–1315 10.1038/nsmb.2417 23142986

[226] Kim S., Lee J. H., Seok J. H., Park Y. H., Jung S. W., Cho A. E., Lee C., Chung M. S., and Kim K. H. (2016) Structural basis of novel iron-uptake route and reaction intermediates in ferritins from Gram-negative bacteria. J. Mol. Biol. 428, 5007–5018 10.1016/j.jmb.2016.10.022 27777002

[227] Lu M., and Fu D. (2007) Structure of the zinc transporter YiiP. Science 317, 1746–1748 10.1126/science.1143748 17717154

